# Synthesis of 1,4-azaphosphinine nucleosides and evaluation as inhibitors of human cytidine deaminase and APOBEC3A

**DOI:** 10.3762/bjoc.20.96

**Published:** 2024-05-15

**Authors:** Maksim V Kvach, Stefan Harjes, Harikrishnan M Kurup, Geoffrey B Jameson, Elena Harjes, Vyacheslav V Filichev

**Affiliations:** 1 School of Food Technology and Natural Sciences, Massey University, Private Bag 11 222, Palmerston North 4442, New Zealand; 2 Maurice Wilkins Centre for Molecular Biodiscovery, Thomas Building of the University of Auckland, Level 2, 3A Symonds Street, Auckland 1142, New Zealand

**Keywords:** APOBEC3, cytidine deaminase, enzyme activity, inhibitor, nucleoside, nucleotide, zebularine

## Abstract

Nucleoside and polynucleotide cytidine deaminases (CDAs), such as CDA and APOBEC3, share a similar mechanism of cytosine to uracil conversion. In 1984, phosphapyrimidine riboside was characterised as the most potent inhibitor of human CDA, but the quick degradation in water limited the applicability as a potential therapeutic. To improve stability in water, we synthesised derivatives of phosphapyrimidine nucleoside having a CH_2_ group instead of the N3 atom in the nucleobase. A charge-neutral phosphinamide and a negatively charged phosphinic acid derivative had excellent stability in water at pH 7.4, but only the charge-neutral compound inhibited human CDA, similar to previously described 2'-deoxyzebularine (*K*_i_ = 8.0 ± 1.9 and 10.7 ± 0.5 µM, respectively). However, under basic conditions, the charge-neutral phosphinamide was unstable, which prevented the incorporation into DNA using conventional DNA chemistry. In contrast, the negatively charged phosphinic acid derivative was incorporated into DNA instead of the target 2'-deoxycytidine using an automated DNA synthesiser, but no inhibition of APOBEC3A was observed for modified DNAs. Although this shows that the negative charge is poorly accommodated in the active site of CDA and APOBEC3, the synthetic route reported here provides opportunities for the synthesis of other derivatives of phosphapyrimidine riboside for potential development of more potent CDA and APOBEC3 inhibitors.

## Introduction

**Figure 1:**
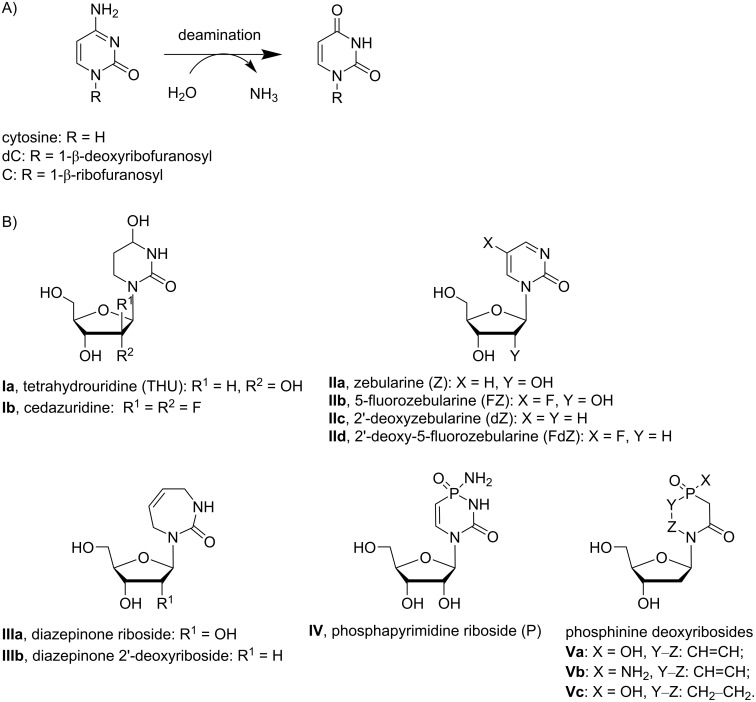
A) Deamination of cytosine, **dC** and **C** as individual nucleosides or as part of a polynucleotide chain. B) Previously described CDA inhibitors and a structure of proposed phosphinine deoxyribosides **Va**–**c**.

Spontaneous hydrolytic deamination of cytosine to uracil (Figure 1A) is very slow under ambient conditions [1], but it is greatly accelerated by enzymes. These enzymes share a similar mechanism of cytosine deamination and a similar tertiary structure. Despite this similarity, individual enzymes are selective for the corresponding cytosine-containing substrates with little or no cross-reactivity. Cytosine deaminase, which is present in bacteria and fungi but not in mammalian cells, acts only on cytosine. Cytidine deaminase (CDA) as a key enzyme in the pyrimidine salvage pathway in mammals deaminates both cytidine and 2'-deoxycytidine. Members of the apolipoprotein B mRNA editing enzyme, catalytic polypeptide-like (APOBEC) family, such as activation-induced deaminase (AID) and APOBEC3 (A3), act preferentially on single-stranded DNA (ssDNA) containing one or multiple cytosine residues. Although some action was detected on RNA, none was observed on cytidine or cytosine alone.

Each cytosine or cytidine deaminase has an important biological function in an organism, but the activities can also be detrimental. CDA is highly active in the liver and spleen, which results in deamination and consequent deactivation of several chemotherapeutic agents, including the anticancer agents cytarabine, gemcitabine and 5-aza-2'-deoxycytidine (decitabine) [2–5]. Full inhibition of CDA leads to accumulation of toxic pyrimidine catabolism intermediates [6–7]. However, local and temporary inhibition of CDA in the liver provides a therapeutic benefit by allowing cytosine analogue-containing drugs to bypass the liver with an intact nucleobase. Recently, a combination of the CDA inhibitor (4*R*)-2′-deoxy-2′,2′-difluoro-3,4,5,6-tetrahydrouridine (cedazuridine, **Ib**, Figure 1B) with the anticancer drug decitabine was approved as an oral pill (i.e., C-DEC or ASTX727) for the treatment of patients with intermediate or high-risk myelodysplastic syndrome (MDS) and chronic myelomonocytic leukaemia (CMML) [8].

In normal human cells, the enzyme family A3 [9–12] disables pathogens by scrambling ssDNA by cytosine to uracil mutation (Figure 1A) [9–10,13–14]. However, several enzymes, particularly A3A, A3B, A3H and A3G, deaminate cytosine in human nuclear and mitochondrial genomes [15]. This A3-induced mutational activity is used by viruses and cancer cells to increase the rates of mutagenesis, which allows them to escape adaptive immune responses and become drug resistant [16–20], leading to poor clinical outcomes. A range of genetic, biochemical and structural studies support a model in which this A3-mediated mutagenesis promotes tumour evolution and strongly influences disease trajectories, including the development of drug resistance and metastasis [18–23]. Of the seven A3 enzymes, three (A3A, A3B and A3H) are at least partially localised in the nucleus of cells and, in cancer cells, become genotoxic [24]. A3A and A3H are single-domain enzymes, whereas A3B is a double-domain enzyme, in which only the C-terminal domain (CTD) has catalytic activity, and the N-terminal domain (NTD) is responsible for binding of DNA and for nuclear localisation.

Initially, A3B had been identified as the primary source of genetic mutations in breast [18–23,25–26] and other cancers [27–28]. The breast cancers with high expression of A3B show a two-fold increase in overall mutational load. Elevated A3B expression correlates with reduced tamoxifen sensitivity of tumours in those patients [19] and poor survival rates for estrogen receptor-positive (ER+) breast cancer patients [21,29]. In line with these observations, A3B overexpression accelerates the development of tamoxifen resistance in murine xenograft with ER+ breast cancer. In contrast, knockdown of A3B results in prolonged tamoxifen responses and leads to the survival of mice for the duration of the experiment (1 year) [19]. More recent research also points at a prominent role of A3A in breast [30] and other cancers [30–33]. Overexpression of A3A and A3B leads to tumorigenesis in transgenic mouse models [24,28,34–35]. High levels of A3A and A3B mRNA are also linked to the more aggressive breast cancers, including triple negative cancers [36]. Since A3B is not essential for humans [37] and A3A does not take part in primary metabolism, inhibition of A3A and A3B offers a potent strategy to suppress cancer evolution and prolong efficacy of existing anticancer therapies [19,38–39].

Despite of the low sequence identity, CDA and A3 share a similar overall structural topology and a close structural homology for the Zn^2+^-containing active site. Since cytosine deamination involves a nucleophilic attack at the C4 position by a Zn^2+^-activated water molecule [40–42], it was proposed to employ transition state analogues and mimetics of the tetrahedral intermediate formed as inhibitors of these enzymes [43–47]. More than 30 compounds have been synthesised in the past and evaluated as inhibitors targeting the active site of CDA. THU (**Ia**) [45,48], zebularine (Z, **IIa**) [47,49–50] and 5-fluorozebularine (FZ, **IIb**) [47,51] as well as diazepinone riboside (**IIIa**) [42–44,52] were among the most potent compounds (Figure 1B). THU (**Ia**) quickly converts into the inactive β-ribopyranosyl form in solution, but substituting hydrogen atoms with fluorine atoms in the 2'-position leads to cedazuridine (**Ib**), which is stable [53] and now used in clinics as a CDA inhibitor in the liver, extending the lifetime of coadministered decitabine [8].

We have recently developed the first rationally designed competitive inhibitors of A3 by incorporating 2'-deoxy derivatives of zebularine, i.e., 2'-deoxyzebularine (dZ, **IIc**) and 5-fluoro-2'-deoxyzebularine (FdZ, **IId**, Figure 1B) [54] as well as diazepinone 2'-deoxyriboside (**IIIb**) [55] into DNA fragments. We demonstrated that dZ (**IIc**) does not inhibit A3 enzymes as the free nucleoside but becomes a low-µM inhibitor if it is used in ssDNA instead of the target dC in the recognition motifs of A3A/A3B and A3G [54]. This observation supports a mechanism in which ssDNA delivers dZ (**IIc**) to the active site for inhibition. By changing the nucleotides around dZ (**IIc**), we obtained the first A3B-selective inhibitor [56]. By inserting the fluoro-substituted FdZ (**IId**) into ssDNA, we observed three times better inhibition of A3Bctd and wild-type A3A in comparison to the **IIc**-containing DNA [57], which correlates with the trend reported for CDA inhibitors [47,51]. We also demonstrated that **IIc**- and **IId**-containing DNAs also inhibit full-length wild-type A3G with similar efficiency to that for the single catalytically active CTD [57–58]. Recently, analysis of crystal structures revealed that both dZ (**IIc**) and FdZ (**IId**) form tetrahedral intermediates after hydrolysis of the N3–C4 double bond in the active sites of A3Gctd and A3A [59–60]. The intermediates formed had the same *R*-stereochemistry at the C4 atom of the nucleobase as previously observed for CDA, and thus confirming the general mechanism of cytosine deamination for A3 and CDA [50,59–64].

The fact that dZ (**IIc**), FdZ (**IId**) and diazepinone 2'-deoxyriboside (**IIIb**) used in the same DNA sequence had a differing inhibitory effect on individual A3 under identical conditions means that the structure of the cytidine analogue determines the inhibitory potential of the inhibitor-containing oligonucleotide [55,57]. This also supports our strategy of using more potent CDA inhibitors in DNA sequences for the development of more powerful A3 inhibitors. The most potent inhibitor of CDA reported so far is phosphapyrimidine riboside (P, **IV**), with an inhibition constant (*K*_i_) of 0.9 nM (Figure 1B) [45]. However, it is unstable in solution and thus cannot be used as CDA inhibitor or incorporated into ssDNA and evaluated as an A3 inhibitor. Here, we report the synthesis of novel inhibitors of CDA and A3 based on the 1,4-azaphosphinine scaffold, compounds **Va**–**c** (Figure 1B), in which the N3 atom present in the nucleobase of **IV** is replaced by CH_2_. We assumed that this change should not significantly affect the inhibitory potential but rather increase the stability of the target nucleosides in water and allow chemical incorporation into ssDNA.

## Results and Discussion

### Synthesis of nucleosides

**Scheme 1:**
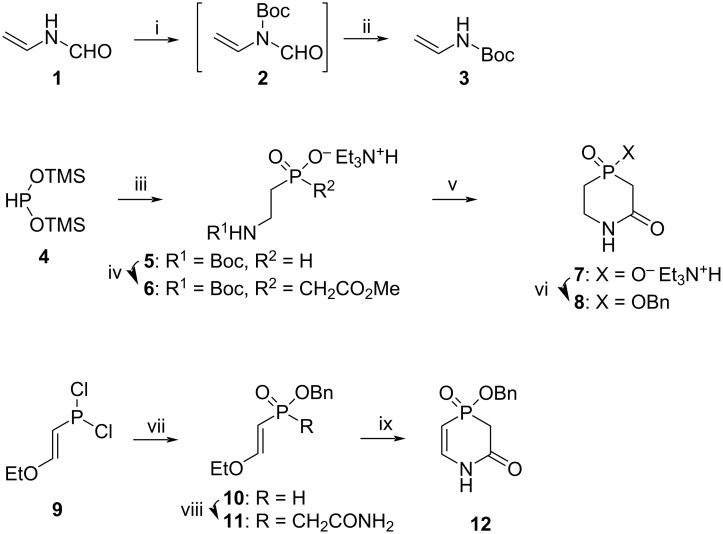
i) Boc_2_O, DMAP, THF, rt, overnight; ii) aq 5 M NaOH, rt, 3 h, 89% yield over two steps; iii) **3**, azobis(isobutyronitrile) (AIBN), ACN, rt to 80–90 °C, overnight, under Ar, followed by Et_3_N/MeOH workup, rt, 50% yield; iv) methyl chloroacetate, Et_3_N, TMSCl, CH_2_Cl_2_, rt, 5 d, 84% yield; v) trifluoroacetic acid, CH_2_Cl_2_, rt, overnight, then 5 h reflux in pyridine/Et_3_N, 84% yield; vi) BnOH, *O*-(benzotriazol-1-yl)-*N*,*N*,*N*′,*N*′-tetramethyluronium tetrafluoroborate (TBTU), Et_3_N, DCE, reflux, 3 h, 65% yield; vii) BnOH, absolute Et_2_O, pyridine, −78 to 0 °C, followed by H_2_O/pyridine workup, 90% yield based on purity of **10** as determined by ^31^P NMR; viii) chloroacetamide, HMDS, ACN, 70 °C, 48 h, under argon, 47% yield; ix) trifluoroacetic acid, CH_2_Cl_2_, rt, overnight, 68% yield.

It is more straightforward to start the synthesis of a modified nucleoside from the assembly of a nucleobase that can be coupled to the sugar afterwards using the Hilbert–Johnson reaction or a silyl variation of it as described in the literature [65]. Scheme 1 shows the synthesis of the target nucleobases.

*N*-Boc-vinylamine (**3**) was synthesised from commercially available *N*-vinylformamide (**1**) as a stable source of vinylamine by treatment of **1** with Boc_2_O in THF in the presence of a catalytic amount of DMAP, followed by cleavage of the formyl moiety under basic conditions. Compound **3** was obtained nearly on a 20 g scale in 89% yield after purification by sublimation in vacuo. In the presence of a catalytic amount of AIBN, compound **3** reacted with bis(trimethylsiloxy)phosphine (**4**) that was prepared in situ [66]. Treatment of the reaction mixture with MeOH/Et_3_N, followed by silica gel column chromatography, led to the triethylammonium salt of 2-*N*-Boc-aminoethylphosphinic acid **5** in 50 % yield. Alkylation of acid **5** with methyl chloroacetate in the presence of TMSCl and Et_3_N took five days at room temperature, and compound **6** as triethylammonium salt was obtained in 84% yield after silica gel purification. Removal of the Boc protecting group from **6** in the presence of trifluoroacetic acid in DCM at room temperature overnight, followed by cyclisation in boiling pyridine/triethylamine, led to 4-hydroxy-1,4-azaphosphinan-2,4-dione (**7**) in 84% yield. The free phosphinic acid **7** was further protected with benzyl alcohol by a procedure adopted from reference [67] using TBTU and Et_3_N in refluxing dichloroethane. Compound **8** was obtained in 65% yield after silica gel column chromatography.

To synthesise a nucleobase for nucleosides **Va** and **Vb**, we first obtained dichlorophosphane **9** from commercially available PCl_3_ and ethyl vinyl ether using a previously published procedure [68]. Compound **9** reacted with 1 equiv of benzyl alcohol in absolute Et_2_O and pyridine at −78 °C, followed by quenching of the reaction mixture with H_2_O. This procedure provided phosphinate **10** in more than 90% purity, as determined by ^31^P NMR. Compound **10** was used in the next step without further purification. A linear amide **11** was obtained in 47% yield by reacting phosphinate **10** with chloroacetamide in the presence of a large excess of HMDS in acetonitrile at 70 °C for two days. A cyclisation of the linear amide **11** was performed in DCM using a 10-fold excess of trifluoroacetic acid at room temperature, providing 1,4-azaphosphinine **12** in 68% yield.

**Scheme 2:**
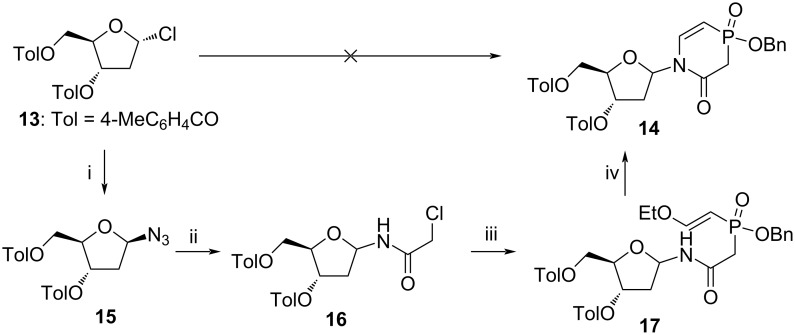
i) NaN_3_, *n*-Bu_4_NHSO_4_, NaHCO_3_/CHCl_3_ (1:1), rt, 20 min, 88% yield; ii) a) H_2_, Pd/C, CH_2_Cl_2_, rt, 3 h; b) chloroacetyl chloride, Et_3_N, 0 °C, overnight, 38% yield; iii) **10**, HMDS, DCE, 90 °C, 24 h, 32% yield; iv) TMSOTf, ACN, 40 °C, 2.5 h, 64% yield.

Various conditions used for the coupling of nucleobase **8**, such as using silylated derivatives (HMDS, BSA) or salts obtained by base treatment (NaH, *t*-BuOK), with Hoffer’s chlorosugar (**13**) in the presence or absence of Lewis acid (TMSOTf, SnCl_4_) did not result in formation of a reasonable amount of appropriate nucleoside (Scheme 2). Nucleobase **12** could not be converted to the corresponding silylated derivative by using HMDS, TMSCl or a combination of both. Difficulties in the Hilbert–Johnson reaction and the low yield observed for nucleoside **14** prompted us to use an alternative option for the synthesis of the target nucleosides based on the assembly of a nucleobase on the 2-deoxyribofuranos-1-yl scaffold.

Hydrogenation of azide **15** [69], followed by treatment of 2-deoxyribofuranosylamine formed in situ with chloroacetyl chloride and Et_3_N, led to 2-deoxyribofuranosyl 2-chloroacetamide **16** in 38% yield with a β/α ratio of about 1:1 (Scheme 2). Phosphinate **10** was then alkylated with compound **16** in the presence of HMDS at elevated temperature, providing a linear nucleoside **17** as a mixture of two anomers, which were successfully separated on a silica gel column. Finally, cyclisation of a linear nucleoside was accomplished in the presence of a catalytic amount of the Lewis acid TMSOTf in 64% yield. Unfortunately, cyclisation was accompanied by racemisation, and nucleoside **14** with the same α/β ratio of 3:2 formed from either anomerically pure **17** or from a mixture of the anomers.

**Scheme 3:**
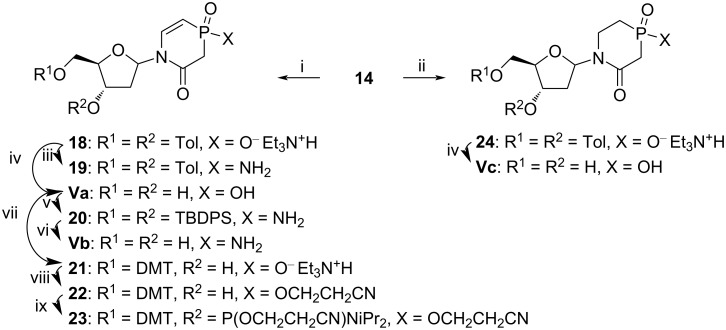
i) H_2_, 5% Pd/CaCO_3_/3% Pb, Et_3_N, CH_2_Cl_2_, rt, 1.5 h, 34% and 21% yield for α- and β-anomer of **18**, respectively; ii) H_2_, 10% Pd/C, Et_3_N, CH_2_Cl_2_, rt, overnight, 23% and 19% yield for α- and β-anomer of **24**, respectively; iii) oxalyl chloride, CHCl_3_, rt, 15 min, then sat. NH_3_ in CHCl_3_, rt, 10 min, 46% yield; iv) 28% aq NH_4_OH, rt, overnight, 78% and 92% yield for α- and β-anomer of **Va** from α- and β-anomer of **18**, respectively, and 39% yield for anomeric mixture of **Vc** from **24**; v) *tert*-butyldiphenylsilyl chloride (TBDPSCl), Et_3_N, CHCl_3_, reflux, 2 h, then oxalyl chloride, CHCl_3_, rt, 30 min, followed by sat. NH_3_ in CHCl_3_, rt, 10 min, 14% yield; vi) *n*-Bu_4_NF·3H_2_O, THF, rt, 1 h, 31% yield; vii) 4,4'-dimethoxytrityl chloride (DMTCl), dry pyridine, rt, overnight, 25% and 20% yield for α- and β-anomer of **21**, respectively, from **14**; viii) 3-hydroxypropionitrile, TBTU, Et_3_N, CH_2_Cl_2_, reflux, 1 h, 27% and 73% yield for α- and β-anomer of **22** from α- and β-anomer of **21**, respectively; ix) *N*,*N*-diisopropylamino-2-cyanoethoxychlorophosphine (CEPCl), Et_3_N, dry CH_2_Cl_2_, rt, 1 h, 44% and 58% yield for α- and β-anomer of **23** from α- and β-anomer of **22**, respectively.

Catalytic hydrogenation is usually used for the removal of benzyl protecting groups. However, standard hydrogenation conditions using 10% Pd/C led to reduction of the C=C double bond in the nucleobase, providing nucleoside **24** (Scheme 3). To circumvent this problem, we used poisoned Pd catalyst (Lindlar’s catalyst, 5% Pd/CaCO_3_/3% Pb) and obtained the desired nucleoside **18**. Individual anomers of nucleosides **18** and **24** were separated on a C18 column using a gradient of CH_3_CN in H_2_O. Removal of toluoyl groups was accomplished in aq NH_3_, providing pure α- and β-nucleoside of **Va** and **Vc**, respectively, carrying a negatively charged phosphinic acid group. These compounds were found to be stable in sodium phosphate buffer at pH 7.0 as no decomposition was observed in NMR samples for several days.

To synthesise the charge-neutral nucleoside **Vb** as shown in Figure 1, the phosphinic acid **18** was converted to the phosphinic chloride, followed by ammonolysis in CHCl_3_ (Scheme 3). The resulting toluoyl-protected compound **19** was obtained in 46% yield but was found to be unstable in the basic medium required to remove the toluoyl groups in the next step. This unfortunate instability of nucleoside **19** in basic medium repelled us from the idea of introducing the charge-neutral compound **Vb** into DNA because basic conditions are used for DNA cleavage and deprotection. To obtain **Vb** for experiments with human CDA (hCDA), we used deprotected nucleoside **Va** as a mixture of anomers and converted it to **Vb** through a four-step one-pot synthesis involving silylation, treatment with oxalyl chloride, ammonolysis and removal of silyl groups. Purified phosphinamide **Vb** was obtained as a mixture of anomers with a α/β ratio of 2:1, as determined by ^1^H and ^13^C NMR.

Deprotected nucleosides **Va** and **Vb** but not **Vc** exhibited absorbance in the UV region with ε_258_ = 4230 L⋅mol^−1^⋅cm^−1^ and ε_262_ = 4730 L⋅mol^−1^⋅cm^−1^, respectively. This was most likely a result of the presence of a double bond next to the P=O unit in nucleosides **Va** and **Vb**, whereas there is no double bond in the nucleobase of compound **Vc**.

For incorporation of nucleoside **Va** into DNA, it needed to be equipped with standard 5'-*O*-DMT and 3'-*O-N*,*N*-diisopropylamino-2-cyanoethoxyphosphanyl groups. Further, the negative charge on the nucleobase needed to be eliminated as it might otherwise interfere with automated DNA synthesis. Starting from compound **14** as a mixture of anomers, compound **Va** was obtained using above described steps, and after installation of a 5'-*O*-DMT group, individual anomers of **21** were isolated on reversed-phase column (C18 medium). Then, the α- or β-anomer of salt **21** was converted to 2-cyanoethoxy derivative **22** using 3-hydroxypropionitrile and TBTU. This was further transformed into the required phosphoramidite **23** as individual α- or β-anomer, which was used in the preparation of modified DNA sequences on a DNA synthesiser.

### Evaluation of 1,4-azaphosphinine derivatives as inhibitors of hCDA, engineered A3B and wild-type A3A

#### Evaluation of hCDA inhibition

We monitored the hCDA-catalysed deamination of dC at 286 nm [70] and analysed the kinetic profiles at various inhibitor concentrations using a global regression analysis of the kinetic data using Lambert’s W function [71]. This method provides better estimates for the Michaelis–Menten constant (*K*_M_) and maximum velocity (*V*_max_) than nonlinear regression analysis of the initial rate (*V*_0_). It is also superior to any of the known linearised transformations of the Michaelis–Menten equation, such as Lineweaver–Burk, Hanes–Woolf and Eadie–Hofstee transformations [71]. Then, *K*_M_ for the substrate and *K*_i_ for each inhibitor were calculated, assuming competitive nature of the inhibitors (Table 1).

**Table 1:** *K*_M_ of the substrate dC and *K*_i_ of dZ (**IIc**) and 1,4-azaphosphinine nucleosides against hCDA.

inhibitor	pH	*K*_m_ of dC (μM)^a^	*K*_i_ (μM)	*K*_m_/*K*_i_

dZ (**IIc**)	7.4	260 ± 40	10.7 ± 0.5	24
β-anomer of **Vb**^b^	7.4	240 ± 150	8.0 ± 1.9	30
β-anomer of **Va**	7.4	—	no inhibition	—
dZ (**IIc**)	6.0	270 ± 60	49 ± 13	5.5
β-anomer of **Va**	6.0	—	no inhibition	—
β-anomer of **Va**	4.7	90 ± 20	560 ± 100	—

^a^*K*_M_ was fitted in each experiment independently (see Supporting Information File 1). ^b^Concentration of β-anomers in solutions was determined by NMR (see Supporting Information File 1) and used as inhibitor concentration, assuming that α-anomers were not inhibiting hCDA.

Initially, we performed this assay in 50 mM sodium phosphate buffer at pH 7.4 (25 °C) and observed that the β-anomer of charge-neutral nucleoside **Vb** exhibited similar inhibition of hCDA as the control dZ (**IIc**). Presence of a negative charge in nucleoside **Va** led to lack of inhibition at pH 7.4. We assumed that protonation of **Va** might result in some inhibition of hCDA. However, the p*K*_a_ of **Va** was estimated to be ≤1.5 (see Supporting Information File 1). This means that the pH value of the assay should be close to pH 1.5 to see any meaningful effect of partially protonated compound **Va**, but hCDA would be denatured at this pH value. By lowering the pH value to 6.0, dZ (**IIc**) started to lose potency against hCDA (Table 1), which might be a result of protonation of the pyrimidine ring in dZ (**IIc**). Some inhibition of hCDA by the β-anomer of **Va** was observed at pH 4.7, with a *K*_i_ value of 560 μM. At this pH value, less than 1 in 1,000 molecules of **Va** might be protonated, which could mean that protonated acid **Va** is a potent hCDA inhibitor.

#### Evaluation of inhibitors against engineered A3A mimic and wild-type A3A by ^1^H NMR-based assay

**Figure 2:**
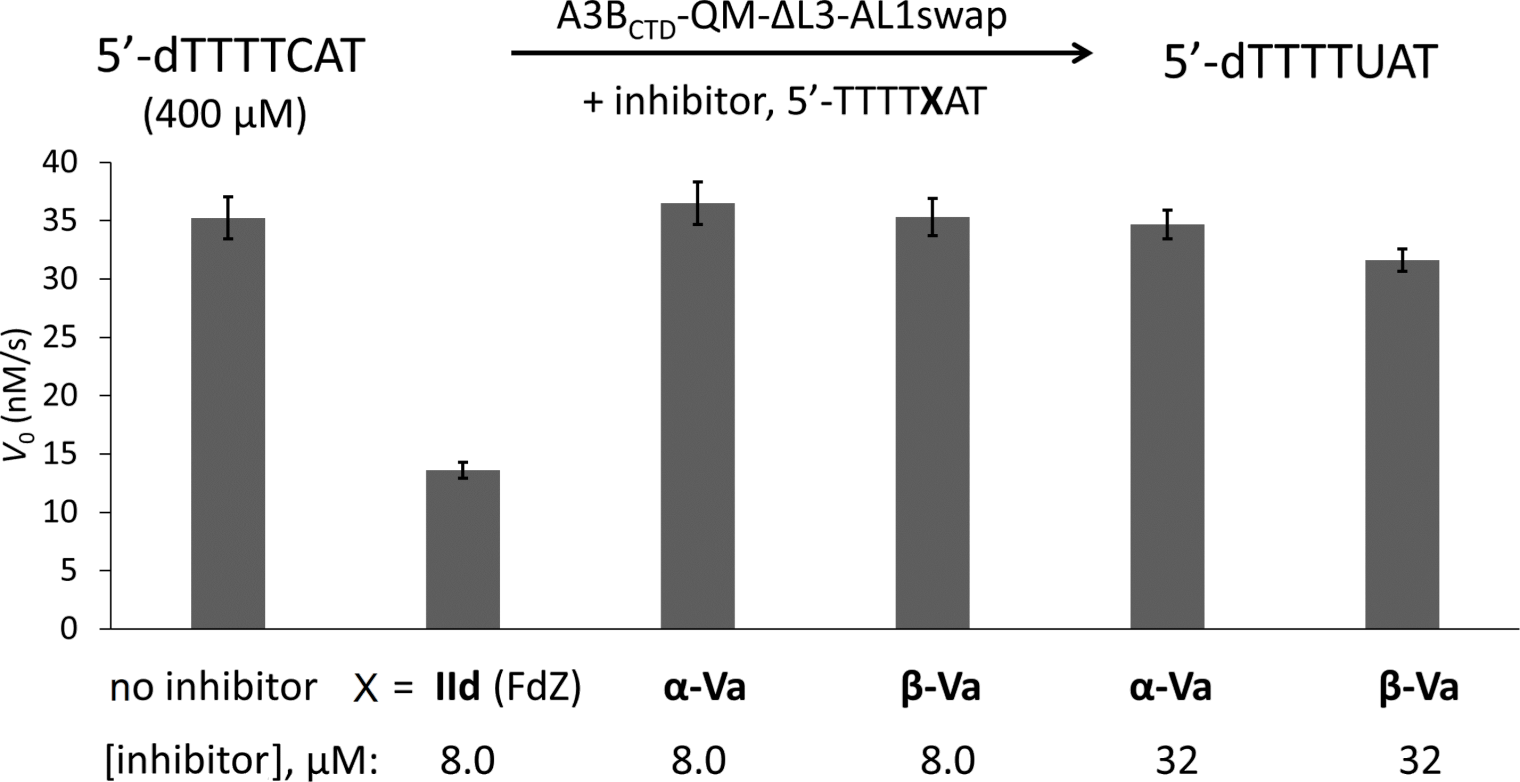
*V*_0_ of A3A mimic-catalysed deamination of 5'-dTTTTCAT in the absence (no inhibitor) and presence of inhibitor at the concentration indicated. Conditions: 400 µM of the substrate 5'-dTTTTCAT, 8 µM or 32 µM of ODN containing α- or β-anomer of **Va** and 8 µM of FdZ (**IId**)-containing ODN (control), 300 nM of A3A mimic in a 50 mM sodium phosphate buffer (pH 6.0) containing 100 mM NaCl, 2.5 mM β-mercaptoethanol, 50 µM 3-(trimethylsilyl)-2,2,3,3-tetradeuteropropionic acid (TSP) and 20% D_2_O at 25 °C. Error bars are estimated standard deviation from triplicate measurements. 5'-dTTTTUAT is the product of the enzymatic reaction.

In a manner analogous to that described in reference [55], we used a ^1^H NMR-based assay to test the short oligodeoxynucleotides (ODNs), linear and hairpins, containing individual α- and β-anomers of nucleoside **Va** as inhibitors of A3. This real-time NMR-based assay is a direct assay: it uses only A3 enzymes and ODNs in a buffer, unlike many fluorescence-based assays where a secondary enzyme and a fluorescently modified oligonucleotide are used [72]. The NMR-based assay provides the initial velocity of deamination of ssDNA substrates, including the modified ones [56], in the presence of A3 enzymes. Consequently, the Michaelis–Menten kinetic model can be used to characterise substrates and inhibitors of A3. Both anomers of the nucleoside **Va** were individually incorporated instead of the target dC in the preferred DNA motif TCA of A3A and A3B on linear DNA. The previously described A3 inhibitor 5'-TTTT**FdZ**AT was used as a control [54,56–57]. The engineered A3A mimic was used in our initial experiments. This is a well-characterised and active derivative of the CTD of A3B (A3B_CTD_), originally called A3B_CTD_-QM-∆L3-AL1swap [54], in which loop 3 is deleted and loop 1 is replaced with the corresponding loop 1 from A3A. The residual activity of the A3A mimic on the unmodified oligonucleotide 5'-TTTTCAT as a substrate in the presence of a known concentration of inhibitor was measured using the NMR-based assay (Figure 2).

The results revealed that both anomers of **Va** do not inhibit engineered A3A mimic even at elevated concentration in comparison to a control ODN containing FdZ (**IId**) at pH 6.0. It is very likely that a negative charge in nucleobase **Va** prevents binding to the enzyme.

Recently, it was reported that A3A prefers deaminating cytosine present in the short loops of DNA hairpins rather than linear DNA at pH 7 [73–75]. We assumed that placing the β-anomer of nucleoside **Va** in a more preferred substrate would allow us to detect inhibitory potential of the resulting DNA hairpin. The β-anomer of **Va** was introduced instead of the target dC in the DNA hairpin with TTC loop and tested in the ^1^H NMR-based assay monitoring A3A-catalysed deamination of dC hairpin (T(GC)_2_TT**C**(GC)_2_T, wherein **C** is deaminated) at a 150 mM salt concentration at pH 7.4. Recently, FdZ (**IId**), 5-methyl-2'-deoxyzebularine and diazepinone 2'-deoxyriboside (**IIIb**) inserted into loops of DNA hairpins have shown selective inhibition of A3A with a half-maximal inhibitory concentration (IC_50_) and *K*_i_ in the low-nM range [55,60,76–77]. Unfortunately, no inhibition of A3A by the DNA hairpin carrying the β-anomer of **Va** was detected at the concentration used (20 and 100 µM of inhibitor DNA, 1 mM dC hairpin as a substrate, 600 nM of wild-type A3A containing His_6_ tag (wtA3A-His_6_) in 50 mM Na^+^/K^+^ phosphate buffer, supplemented with 100 mM NaCl, 1 mM tris(2-carboxyethyl)phosphine (TCEP), 100 µM sodium trimethylsilylpropanesulfonate (DSS) and 10% D_2_O at pH 4.7).

## Conclusion

Nucleoside and polynucleotide (A3) CDA share a universal mechanism of target nucleobase engagement, deamination and inhibition [50,59–64]. We have recently demonstrated the first inhibition of A3A-induced mutagenesis in cells using a DNA hairpin carrying FdZ (**IId**) instead of the target C in the TTC loop [60]. To further improve potency of DNA-based inhibitors of A3, more potent inhibitors of cytosine deamination than previously characterised dZ (**IIc**), FdZ (**IId**) and diazepinone 2'-deoxyriboside (**IIIb**) can be used. There are two obvious choices based on the literature on CDA inhibitors, THU (**Ia**) and phosphapyrimidine nucleoside **IV** (Figure 1). However, the hemiaminal functionality in the nucleobase and the fast transformation into pyranose in THU (**Ia**) along with instability of nucleoside **IV** in water prevent the incorporation into DNA fragments using conventional DNA synthesis chemistry. Here, we hypothesised that the aqueous stability of **IV** could be significantly improved by changing the N3 atom in the nucleobase to a methylene group, providing nucleosides **Va**–**c** with and without a double bond between the C5 and C6 atoms (Figure 1). Towards this end, we developed a synthetic strategy for these nucleosides and identified that assembly of the nucleobase on the sugar was more viable than coupling of the final nucleobase to Hoffer’s chlorosugar (**13**). It is interesting that only the charge-neutral phosphinamide **Vb** inhibited hCDA similarly to dZ (**IIc**) at pH 7.4, whereas negatively charged phosphinic acid **Va** showed some inhibition of hCDA only at pH 4.7. Unfortunately, due to the low stability of charge-neutral phosphinamide **Vb** towards nucleophiles, we could not incorporate it into DNA. Synthesis of a DMT-protected phosphoramidite of nucleoside **Va** and the incorporation into DNA was more straightforward, but no inhibition of A3A was observed for these ODNs. These results suggest that negatively charged nucleobases cannot be accommodated in the active site of hCDA and A3A, and other options need to be considered for the development of new nucleobases mimicking transitions states and an intermediate of cytosine deamination to improve potency of DNA-based A3 inhibitors.

## Supporting Information

File 1 Supplementary experimental details about the enzymatic assays and the synthesis of nucleosides and modified ODNs, assignment of ^1^H, ^13^C, ^31^P NMR and IR spectra and results of HRESIMS experiments for new compounds synthesised as well as RP-HPLC profiles and HRESIMS spectra of ODNs.

## Data Availability

The data that supports the findings of this study is available from the corresponding author upon reasonable request.
