# Supplementary material for: Synthesis of 1,4-azaphosphinine nucleosides and evaluation as inhibitors of human cytidine deaminase and APOBEC3A
Source: Beilstein J Org Chem. 2024 May 15;20:1088–98. doi: 10.3762/bjoc.20.96 (PMC11106675; doi:10.3762/bjoc.20.96)
Supplement: File 1 — Supplementary experimental details about the enzymatic assays and the synthesis of nucleosides and modified ODNs, assignment of 1H, 13C, 31P NMR and IR spectra and results of HRESIMS experiments for new compounds synthesised as well as RP-HPLC profiles and HRESIMS spectra of ODNs. [file Beilstein_J_Org_Chem-20-1088-s001.pdf]

BEILSTEIN JOURNAL OF ORGANIC CHEMISTRY

Supporting Information

for

Synthesis of 1,4-azaphosphinine nucleosides and evaluation as inhibitors of human cytidine deaminase and APOBEC3A

Maksim V. Kvach, Stefan Harjes, Harikrishnan M. Kurup, Geoffrey B. Jameson, Elena Harjes and Vyacheslav V. Filichev

Beilstein J. Org. Chem. **2024,** *20,* 1088–1098. doi:10.3762/bjoc.20.96

Supplementary experimental details about the enzymatic assays and the synthesis of nucleosides and modified ODNs, assignment of ^{1}H, ^{13}C, ^{31}P NMR and IR spectra and results of HRESIMS experiments for new compounds synthesised as well as RP-HPLC profiles and HRESIMS spectra of ODNs

License and Terms: This is a supporting information file under the terms of the Creative Commons Attribution License (https://creativecommons.org/licenses/by/4.0). Please note that the reuse, redistribution and reproduction in particular requires that the author(s) and source are credited and that individual graphics may be subject to special legal provisions.
The license is subject to the *Beilstein Journal of Organic Chemistry* terms and conditions: (https://www.beilstein-journals.org/bjoc/terms)

Table of Contents

General methods. S2
Synthetic procedures. S2
Synthesis of modified oligonucleotides S22
Protein expression and purification..S26
UV kinetic assay of hCDA-catalysed 2'-deoxycytidine deamination S26
^{1}H NMR-based kinetic assay of A3-catalysed deamination S29
References S29

General methods

Reagents from commercial suppliers were used without further purification. Pyridine and CH_2Cl_2 were freshly distilled from CaH_2, and Et_2O, ethyl vinyl ether, THF and Et_3N were distilled over Na. Hoffer's chlorosugar (**13**) was prepared as previously described [1]. Saturated NH_3 in $CHCl_3$ was prepared by bubbling NH_3 gas in ice-cooled $CHCl_3$ until saturation, and the solution was stored at 4 °C. ^{1}H (400, 500 or 700 MHz), ^{13}C (100.6, 125.8 or 176.1 MHz) and ^{31}P (202.5 MHz) NMR spectra were obtained with Bruker Avance 400, Avance 500 or Avance 700 MHz spectrometers and referenced to residual solvent signals ($CDCl_3$: 7.26 ppm for ^{1}H and 77.16 ppm for ^{13}C; DMSO- *d*$_6$: 2.50 ppm for ^{1}H and 39.52 ppm for ^{13}C, according to Reference [2]; 3-(trimethylsilyl)-2,2,3,3-tetradeuteropropionic acid (TMSP-*d*$_4$): 0.0 ppm for ^{1}H and ^{13}C, internal standard for water samples, or 85% aq. H_3PO_4 external standard: 0.0 ppm for ^{31}P. ^{1}H NMR coupling constants are reported in Hertz (Hz) and refer to apparent multiplicities. The assignment of signals was done using 2D homonuclear ^{1}H,^{1}H-COSY, NOESY and heteronuclear ^{1}H,^{13}C-HMQC or HSQC, and HMBC spectra. NMR spectra were processed in Spinworks version 4.1 (developed by Dr. Kirk Marat, Department of Chemistry, University of Manitoba). High-resolution electrospray mass spectra were recorded on a Thermo Scientific Q Exactive Focus Hybrid Quadrupole-Orbitrap mass spectrometer. Ions generated by ESI were detected in positive or negative ion modes. Total ion count (TIC) was recorded in centroid mode over the *m*/*z* range of 100–3,000 and analyzed using ThermoXcalibur Qual Browser. Analytical thin-layer chromatography was performed on Kieselgel 60 F_{254} precoated aluminum plates (Merck). Spots were visualised by UV or stained with I_2/SiO_2, aq $KMnO_4$, ninhydrin solution or cerium ammonium molybdate (CAM) stain. The latter was prepared by suspending $Ce(NH_4)_4(SO_4)_4 \cdot 2H_2O$ (4 g) and ammonium molybdate (10 g) in H_2O (360 mL), followed by addition of 98% H_2SO_4 (40 mL). Silica gel column chromatography was performed on silica gel 60 with size particles 40–63 µm or 20–45 µm (specified) using Büchi Sepacore X50 flash chromatography system. Oligonucleotide synthesis was carried out on a MerMade-4 DNA/RNA synthesizer (BioAutomation) on a 5 µmol scale using standard manufacturer's protocol. "Saltless buffer" used for oligonucleotide desalting consisted of 10 µM Tris-HCl (pH 8.0), 1 µM EDTA and 0.001% w/v NaN_3.

Synthetic procedures

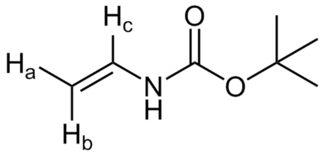

N-Boc-vinylamine (**3**) was prepared starting from commercially available *N*-vinylformamide (**1**) as a stable source of vinylamine. To a solution of *N*-vinylformamide (**1**, 10.5 mL, 150 mmol) and Boc_2O (36.0 g, 165 mmol) in THF (150 mL), DMAP (183 mg, 1.5 mmol) was added, and the reaction mixture was stirred overnight at room temperature. An aliquot of the reaction mixture was evaporated, and the progress of the reaction was controlled by ^{1}H NMR, which clearly showed formation of *N*-Boc-*N*-vinylformamide (**2**) as a major product.

^{1}H NMR (500 MHz, *d*$_6$-DMSO) δ 9.16 (s, 1H, C*H*O), 6.56 (ddd, 1H, $J_{Hb,Hc}$ = 16.2 Hz, $J_{Ha,Hc}$ = 9.6 Hz, J = 0.9 Hz, H_c), 5.56 (d, 1H, $J_{Hb,Hc}$ = 16.2 Hz, H_b), 5.04 (dd, 1H, $J_{Ha,Hc}$ = 9.6 Hz, J = 0.9 Hz, H_a), 1.51 (s, 9H, CH_3).

To cleave the formyl group, an aqueous solution of NaOH (90 mL, 5 M) was added, and the mixture was stirred for 3 h. The water layer was separated, extracted with AcOEt (2 × 150 mL), the combined organic layers were washed with brine (150 mL), dried over Na_2SO_4, filtered and evaporated. *N*-Boc-vinylamine (**3**) was purified by sublimation in vacuo (1 mmHg), resulting in 19.1 g (89%) of white solid.

^1H NMR (400 MHz, d_6-DMSO) δ 9.20 (d, 1H, J = 9.4 Hz, NH), 6.51 (m, 1H, H_c), 4.45 (d, 1H, $J_{Hb,Hc}$ = 15.8 Hz, H_b), 4.09 (d, 1H, $J_{Ha,Hc}$ = 8.8 Hz, H_a), 1.41 (s, 9H, CH_3).
$^{13}C\{^1H\}$ NMR (100.6 MHz, d_6-DMSO) δ 152.9 (CO), 130.8 (CH), 92.0 (CH$_2$), 79.0 (C(CH$_3$)$_3$), 28.0 (CH$_3$).

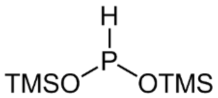

Bis(trimethylsilyloxy)phosphine (BTSP, **4**) was prepared as previously described [3]. A mixture of ammonium hypophosphite (20.75 g, 250 mmol) with hexamethyldisilazane (HMDS, 62.4 mL, 300 mmol) was heated at 100–110 ºC for 2.5–3 h under argon atmosphere. The resulting homogenous liquid of **4** was cooled to room temperature under argon and used without purification.

(Caution! BTSP is highly pyrophoric in air, and thus all operations must be performed under argon).

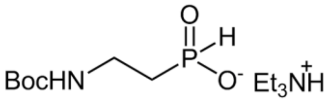

2-*N*-Boc-aminoethylphosphinic acid, triethylammonium salt (**5**). A solution of *N*-Boc-vinylamine (**3**, 14.30 g, 100 mmol) and azobis(isobutyronitrile) (AIBN, 3.28 g, 20 mmol) in acetonitrile (200 mL) was added to BTSP (prepared from ammonium hypophosphite (20.75 g, 250 mmol) and HMDS (62.4 mL, 300 mmol) at room temperature. The reaction mixture was stirred at 80–90 ºC (oil bath) overnight under argon, quenched with a mixture of Et_3N (42.5 mL) and MeOH (25 mL), the solids were filtered out and the filtrate was evaporated in vacuo. Column chromatography on silica eluting with H_2O/iPrOH (5:95, v/v) resulted in a mixture of 2-*N*-Boc-aminoethylphosphinic acid along with the triethylammonium salt **5**. Coevaporation with Et_3N/CH_2Cl_2 led to triethylammonium salt **5** (15.6 g, 50%).

R_f 0.34 (MeOH/Et_3N/CH_2Cl_2, 7:5:88, v/v/v), stains with ninhydrin.
^1H NMR (500 MHz, d_6-DMSO) δ 6.90 (d, 1H, $^1J_{P,H}$ = 484.2 Hz, PH), 6.82 (m, 1H, NH), 3.08 (m, 2H, CH_2NH), 3.00 (d, 6H, J = 6.9 Hz, CH_2CH$_3$), 1.50 (m, 2H, CH$_2$CH_2P), 1.36 (s, 9H, Boc), 1.17 (t, 9H, J = 7.2 Hz, CH$_2$CH_3).
$^{13}C\{^1H\}$ NMR (125.7 MHz, d_6-DMSO) δ 155.3 (CO), 77.6 ((CH$_3$)$_3$$C$), 45.2 (3C, CH$_2CH_3$), 34.0 (CH$_2$NH), 31.9 (d, $^1J_{C,P}$ = 88.9 Hz, CH$_2$$CH_2$P), 28.2 (3C, ($CH_3$)$_3$C)), 8.4 (3C, CH$_2$$CH_3$).
$^{31}P\{^1H\}$ NMR (202.4 MHz, d_6-DMSO, extn. ref. 85% H_3PO_4) δ 20.2.
HRMS (ESI -): calcd. for $C_7H_{15}NO_4P^-$ [M-Et_3NH]$^-$ 208.0744; found 208.0752.

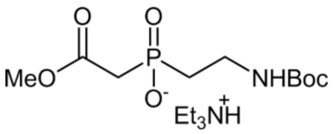

(2-*N*-Boc-aminoethyl)(2-methoxy-2-oxoethyl)phosphinic acid, triethylammonium salt (**6**) was prepared from compound **5** by alkylation with methyl chloroacetate. To a solution of phosphinic salt **5** (3.89 g, 12.5 mmol), Et_3N (3.48 mL, 25.0 mmol) and TMSCl (3.17 mL, 25.0 mmol) in CH_2Cl_2 (25 mL) and methyl chloroacetate (1.65 mL, 18.8 mmol) were added. The mixture was stirred under argon for 5 days at room temperature, quenched with MeOH (2 mL, heated until reflux) and volatiles were removed in vacuo. The residue was purified on silica gel, eluting with H_2O/iPrOH 5:95 (v/v), resulting in alkylated salt **6** (4.04 g, 84%) after coevaporation with Et_3N/CH_2Cl_2.

R_f 0.52 (MeOH/Et_3N/CH_2Cl_2, 7:5:88, v/v/v), stains with ninhydrin.
^{1}H NMR (500 MHz, d_6-DMSO) δ 6.88 (m, 1H, N*H*), 3.53 (s, 3H, CH_3O), 3.12 (m, 2H, CH_2NH), 2.96 (q, 6H, J = 7.2 Hz, CH_2CH$_3$), 2.51 (d, 2H, $^2J_{H,P}$ = 16.4 Hz, CH_2CO$_2$Me), 1.58 (m, 2H, CH$_2$CH_2P), 1.36 (s, 9H, Boc), 1.17 (t, 9H, J = 7.2 Hz, CH$_2$CH_3).
^{13}C{^{1}H} NMR (125.7 MHz, d_6-DMSO) δ 169.4 (*C*O$_2$Me), 155.3 (CONH), 77.3 ((CH$_3$)$_3$*C*), 51.3 (O*C*H$_3$), 44.9 (3C, *C*H$_2$CH$_3$), 38.8 (MeO$_2$C*C*H$_2$P, from HMQC), 35.3 (*C*H$_2$NH), 31.2 (d, $^1J_{C,P}$ = 93 Hz, CH$_2$*C*H$_2$P), 28.2 (3C, (*C*H$_3$)$_3$C)), 8.4 (3C, CH$_2$*C*H$_3$).
^{31}P{^{1}H} NMR (202.4 MHz, d_6-DMSO) δ 24.4.
HRMS (ESI -): calcd. for $C_{10}H_{19}NO_6P^-$ [M-Et$_3$NH]$^-$ 280.0955; found 280.0971.

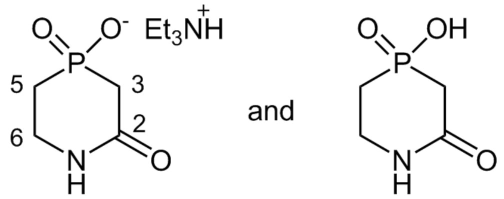

4-Hydroxy-1,4-azaphosphinan-2,4-dione, triethylammonium salt (**7**). To a solution of phosphinic salt **6** (3.89 g, 10.2 mmol) in CH_2Cl_2 (10 mL), trifluoroacetic acid (10 mL) was added, and the mixture was stirred at room temperature overnight. All volatiles were evaporated in vacuo, and the residue was dissolved in pyridine (20 mL), followed by the addition of Et_3N (7.1 mL, 51 mmol). The resulting mixture was refluxed 5 h, solvents were evaporated, and the residue was purified using silica gel column chromatography eluting with a step gradient of H_2O (10→50%) in iPrOH. Fractions containing the product were evaporated, coevaporated with H_2O/Et_3N and dried in vacuo, resulting in saturated nucleobase **7** as a triethylammonium salt (2.13 g, 84%).

R_f 0.25 (H_2O/iPrOH, 1:4, v/v), stains with CAM.
^{1}H NMR (500 MHz, D_2O, intn. ref. *d*4-TMSP) δ 3.49 (m, 2H, H-6), 3.20 (q, 6H, J = 7.3 Hz, CH_2CH$_3$), 2.71 (d, 2H, $^2J_{H3,P}$ = 16.0 Hz, H-3), 1.86 (m, 2H, H-5), 1.28 (t, 9H, J = 7.3 Hz, CH$_2$CH_3).
^{13}C{^{1}H} NMR (125.7 MHz, D_2O, *d*4-TMSP) δ 176.5 (C2), 49.6 (3C, *C*H$_2$CH$_3$), 40.4 (d, $^1J_{C3,P}$ = 74.0 Hz, C3), 40.2 (d, $^2J_{C6,P}$ = 5.6 Hz, C6), 29.7 (d, $^1J_{C5,P}$ = 94.3 Hz, C5), 11.2 (3C, CH$_2$*C*H$_3$).
^{31}P{^{1}H} NMR (202.4 MHz, D_2O, extn. ref. 85% H_3PO_4) δ 34.4.
HRMS (ESI -): calcd. for $C_4H_7NO_3P^-$ [M-Et$_3$NH]$^-$ 148.0169; found 148.0171.

An analytical amount of heterocycle **7** was obtained in the form of free acid by passing an aq. solution of the triethylammonium salt through the column with Dowex-50W×8 (H-form) and eluting with water. After evaporation of water, the resulting free acid **7** slowly solidifies at room temperature.
^{1}H NMR (400 MHz, D_2O) δ 3.48 (m, 2H, H-6), 2.85 (d, 2H, $^2J_{H3,P}$ = 16.7 Hz, H-3), 1.99 (m, 2H, H-5).

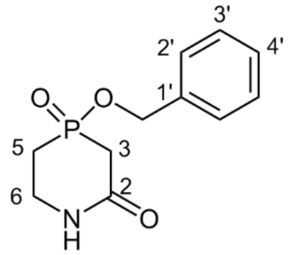

4-(Benzyloxy)-1,4-azaphosphinan-2,4-dione (**8**) was obtained by esterification of phosphinic acid **7** with benzyl alcohol in the presence of *O*-(benzotriazol-1-yl)-*N*,*N*,*N'*,*N'*-tetramethyluronium tetrafluoroborate (TBTU) [4]. Nucleobase **7** as a free acid (1.20 g, 8.05 mmol) was suspended in DCE (32 mL), and TBTU (5.17 g, 16.1 mmol), Et_3N (2.24 mL, 16.1 mmol) and BnOH (1.66 mL, 16.1 mmol) were added. The mixture was refluxed for 3 h until a homogeneous solution was obtained, evaporated in vacuo and purified on silica gel (40 × 75 mm column), eluting with acetone over 30

min with a flow rate of 50 mL/min. Fractions containing the product were evaporated in vacuo to result in oil **8** (1.24 g, 65%), which solidified upon standing.
R_f 0.13 (acetone), UV-active, stains with I_2/SiO_2.
^{1}H NMR (500 MHz, d_6-DMSO) δ 7.88 (br. s, 1H, N*H*), 7.42-7.31 (m, 5H, H-2',3',4'), 5.06-4.97 (m, 2H, CH_2Ph), 3.48-3.20 (m, 2H, H-6), 3.08-2.88 (m, 2H, H-3), 2.12-1.89 (m, 2H, H-5).
^{13}C{^{1}H} NMR (125.8 MHz, d_6-DMSO) δ 167.06 (d, $^2J_{C2,P}$ = 4.5 Hz, C2), 136.6 (d, $^3J_{C1',P}$ = 6.5 Hz, C1'), 127.85 (2C, C2'), 128.2 (C4'), 128.45 (2C, C3'), 65.3 (d, $^2J_{CPh,P}$ = 5.9 Hz, *C*H_2Ph), 35.90 (d, $^1J_{C3,P}$ = 72.5 Hz, C3), 35.57 (d, $^2J_{C6,P}$ = 4.9 Hz, C6), 25.9 (d, $^1J_{C5,P}$ = 92.7 Hz, C5).
^{31}P{^{1}H} NMR (202.5 MHz, d_6-DMSO, extn. ref. 85% H_3PO_4) δ 50.7.
HRMS (ESI +): calcd. for $C_{11}H_{14}NNaO_3P^+$ $[M+Na]^+$ 262.0609; found 262.0603.

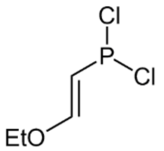

(*E*)-Dichloro(2-ethoxyvinyl)phosphane (**9**) [5] was prepared as follows: To a solution of ethyl vinyl ether (36.0 g, 0.50 mol) and Et_3N (50.5 g, 0.50 mol) in CH_2Cl_2 (250 mL), freshly distilled PCl_3 (43.7 mL, 0.50 mol) was added dropwise, and the mixture was stirred for 2 days at room temperature under argon. The mixture was filtered, solids were washed with hexane, and all solvents were evaporated in vacuo. The residue was stirred with hexane (0.5 L) for 1 h, filtered and evaporated in vacuo. Remaining liquid was distilled in vacuo, resulting in 46.3 g (54 %) of colourless compound **9**.

B.p. 90–100 ºC (30 mbar).
^{1}H NMR (500 MHz, $CDCl_3$) δ 7.11 (dd, 1H, $J_{H,H}$ = 14.4 Hz, $^3J_{H,P}$ = 12.5 Hz, C*H*OEt), 5.79 (1H, $J_{H,H}$ = 14.4 Hz, $^2J_{H,P}$ = 4.6 Hz, C*H*P), 3.97 (q, 2H, *J* = 7.1 Hz, CH_2CH$_3$), 1.36 (t, 3H, *J* = 7.1 Hz, CH$_2$CH_3).
^{13}C{^{1}H} NMR (125.7 MHz, $CDCl_3$) δ 159.6 (d, $^2J_{C,P}$ = 89.3 Hz, *C*HOEt), 108.0 (d, $^1J_{C,P}$ = 46.8 Hz, *C*HOP), 66.4 (*C*H$_2$CH$_3$), 14.3 (CH$_2$*C*H$_3$).
^{31}P{^{1}H} NMR (202.5 MHz, $CDCl_3$, ref. 85% H_3PO_4) δ 167.2.

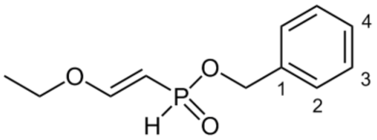

Benzyl (*E*)-(2-ethoxyvinyl)phosphinate (**10**). To a cooled (−78 ºC) solution of dichlorophosphane **9** (52.0 g, 0.30 mol) in absolute Et_2O (300 mL), pyridine (24.2 mL, 0.3 mol), followed by a solution of BnOH (32.5 g, 0.3 mol) in absolute Et_2O (200 mL), were added dropwise over 20 min. The reaction mixture was stirred 1 h at −78 ºC, then heated to 0 ºC and stirred 1 h at 0 ºC. A solution of H_2O (6.50 mL, 0.36 mol) and pyridine (29.0 mL, 0.36 mol) in THF/Et_2O (30/30 mL) was quickly added to the reaction mixture with manual shaking. The mixture was stirred overnight at room temperature, filtered and solids were washed with Et_2O. The combined organic layers were evaporated in vacuo, resulting in phosphinate **10** as yellow oil (67.6 g). Phosphinate **10** was about 90% pure according to ^{31}P NMR and was used without further purification.

R_f 0.31 (acetone/CH_2Cl_2, 1:9, v/v), UV-active, stains with $KMnO_4$.
^{1}H NMR (500 MHz, $CDCl_3$) δ 7.41-7.29 (m, 5H, Ph*H*), 7.30 (d, 1H, $^1J_{H,P}$ = 567.5 Hz, P*H*), 7.18 (dd, 1H, $J_{H,H}$ = 13.7 Hz, $^3J_{H,P}$ = 12.3 Hz, OC*H*), 5.06 (m, 2H, CH_2Ph), 4.79 (dd, 1H, $J_{H,H}$ = 13.7 Hz, $^2J_{H,P}$ = 15.2 Hz, PC*H*), 3.89 (q, 2H, *J* = 7.1 Hz, CH_2), 1.32 (t, 3H, *J* = 7.1 Hz, CH_3).
^{13}C{^{1}H} NMR (125.7 MHz, $CDCl_3$) δ 164.32 (d, $^2J_{C,P}$ = 19.8 Hz, O*C*H), 136.03 (d, $^3J_{C,P}$ = 6.7 Hz, C1), 128.66, 127.96 (4C, C2,3), 128.49 (C4), 90.73 (d, $^1J_{C,P}$ = 142.1 Hz, P*C*H), 66.57 (d, $^2J_{C,P}$ = 5.7 Hz, *C*H$_2$Ph), 66.56 (*C*H$_2$), 14.24 (*C*H$_3$).
^{31}P NMR (202.5 MHz, $CDCl_3$, ref. 85% H_3PO_4) δ 26.17 (dm, $^1J_{H,P}$ = 567.5 Hz).

HRMS (ESI +): calcd. for $C_{11}H_{15}NaO_3P^+$ $[M+Na]^+$ 249.0656; found 249.0651.

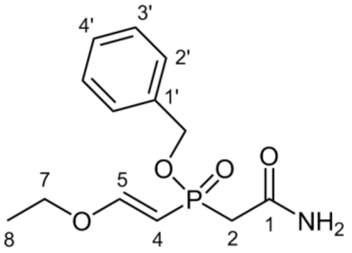

Benzyl (*E*)-(2-amino-2-oxoethyl)(2-ethoxyvinyl)phosphinate (**11**). To a solution of phosphinate **10** (3.84 g, 17.0 mmol) in ACN (17 mL), chloroacetamide (2.07 g, 22.1 mmol), followed by HMDS (11.0 mL, 51 mmol) were added, and the mixture was stirred 48 h at 70 ºC (oil bath) under argon. The reaction was quenched with MeOH (5 mL, stirred for 30 min at 70 ºC), filtered and concentrated in vacuo. The compound was purified by column chromatography on silica gel (40 × 75 mm column), eluting with a gradient of MeOH (0→10%) in CH_2Cl_2 over 7.5 min at a flow rate of 100 mL/min, which resulted in linear amide **11** (2.28 g, 47%).

R_f 0.22 (MeOH/CH_2Cl_2, 5:95, v/v), UV-active, stains with $KMnO_4$.
^{1}H NMR (500 MHz, $CDCl_3$) δ 7.37-7.28 (m, 5H, H-2',3',4'), 7.22 (dd, 1H, $^3J_{4,5}$ = 13.6 Hz, $^3J_{H,P}$ = 11.0 Hz, H-5), 6.88, 5.83 (2br s, 2H, N*H2*), 5.00 (m, 2H, C*H2*Ph), 4.75 (dd, 1H, $^2J_{H,P}$ = 15.8 Hz, $^3J_{4,5}$ = 13.6 Hz, H-4), 3.92-3.79 (m, 2H, H-7), 2.96-2.78 (m, 2H, H-2), 1.30 (t, 3H, $J_{7,8}$ = 7.1 Hz, H-8).
^{13}C{^{1}H} NMR (125.8 MHz, $CDCl_3$) δ 167.1 (C1), 164.4 (d, $^2J_{C5,P}$ = 17.6 Hz, C5), 136.1 (d, $^3J_{C1',P}$ = 6.6 Hz, C1'); 128.6 (2C), 128.4 (1C), 127.8 (2C) (C2',3',4'); 89.4 (d, $^1J_{C4,P}$ = 143.2 Hz, C4), 66.5 (C7), 66.0 (d, $^2J_{C,P}$ = 5.9 Hz, *C*H2Ph), 39.6 (d, $^1J_{C2,P}$ = 93.2 Hz, C2), 14.3 (C8).
^{31}P{1H} NMR (202.5 MHz, $CDCl_3$, extn. ref. 85% H_3PO_4) δ 40.4.
HRMS (ESI +): calcd. for $C_{13}H_{18}NNaO_4P^+$ $[M+Na]^+$ 306.0871; found 306.0866.

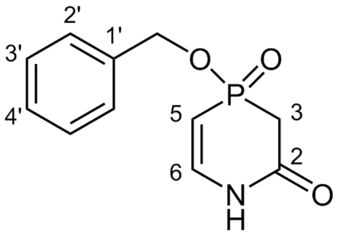

4-(Benzyloxy)-3-hydro-1,4-azaphosphinin-2(1*H*)-one 4-oxide (**12**). To a solution of linear amide **11** (2.83 g, 10.0 mmol) in CH_2Cl_2 (50 mL), trifluoroacetic acid (7.66 mL, 100 mmol) was added, and reaction was stirred overnight at room temperature, evaporated and purified by column chromatography on silica gel (21 × 129 mm column), eluting with a gradient of acetone (0→50%) in CH_2Cl_2 over 20 min at a flow rate of 25 mL/min, which resulted in the cyclic amide **12** (1.60 g, 68%). R_f 0.34 (acetone/CH_2Cl_2, 1:1, v/v).

^{1}H NMR (500 MHz, $CDCl_3$) δ 8.52 (br s, 1H, N*H*), 7.40-7.30 (m, 5H, H-2',3',4'), 6.75 (ddd, 1H, *J* = 39.7 Hz, *J* = 10.8 Hz, *J* = 5.7 Hz, H-6), 5.30 (br dd, 1H, *J* = 10.8 Hz, *J* = 7.0 Hz, H-5), 5.06 (m, 2H, C*H2*Ph), 3.18-2.99 (m, 2H, H-3).
^{13}C{^{1}H} NMR (125.8 MHz, $CDCl_3$) δ 166.8 (d, $^2J_{C2,P}$ = 5.0 Hz, C2), 140.0 (d, $^2J_{C6,P}$ = 2.5 Hz, C6), 135.8 (d, $^3J_{C1',P}$ = 5.7 Hz, C1'); 128.7 (3C), 128.1 (2C) (C2',3',4'); 96.7 (d, $^1J_{C5,P}$ = 127.7 Hz, C5), 66.8 (d, $^2J_{C,P}$ = 6.0 Hz, *C*H2Ph), 37.2 (d, $^1J_{C3,P}$ = 83.8 Hz, C3).
^{31}P{^{1}H} NMR (202.5 MHz, $CDCl_3$, extn. ref. 85% H_3PO_4) δ 32.1.
HRMS (ESI +): calcd. for $C_{11}H_{12}NNaO_3P^+$ $[M+Na]^+$ 260.0452; found 260.0447.

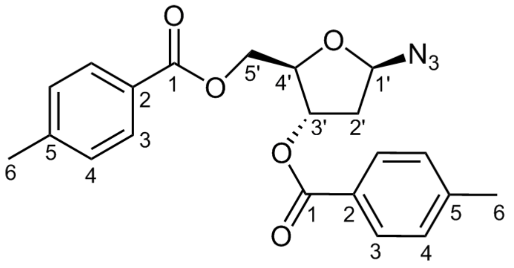

2-Deoxy-3,5-bis[*O*-(4-methylbenzoyl)]-β-D-*erythro*-pentofuranosyl azide (**15**) [6]. To a stirring solution of sodium azide (97.5 g, 1.5 mol) and tetrabutylammonium hydrogen sulfate (101.7 g, 0.3 mmol) in saturated sodium bicarbonate solution (1 L), chloroform (1 L) was added, and the mixture was vigorously stirred for about 5 min until a milky emulsion was formed. Hoffer's chlorosugar (**13**) (116.7 g, 0.3 mol) was rapidly added to the emulsion, and the mixture was stirred 20 min. After the disappearance of starting material, the organic layer was washed with sat. sodium bicarbonate (1 L), water (2 × 1 L), dried over anhydrous sodium sulfate and was filtered. Solvent was evaporated in vacuo, and the product was recrystallized from ethanol (450 mL) to yield compound **2** (105 g, 88%, β:α ratio of 19:1).

R_f 0.47 (CH_2Cl_2).
^{1}H NMR is fully consistent with the data reported in [7]. ^{1}H NMR (500 MHz, d_6-DMSO) δ 7.94-7.84 (m, 4H, H-3), 7.36-7.29 (m, 4H, H-4), 5.879 (dd, 1H, J = 6.0 Hz, J = 4.2 Hz, H-1'), 5.56-5.50 (m, 1H, H-3'), 4.59-4.39 (m, 3H, H-4',5'), 2.49-2.42 (m, 1H, H-2'α), 2.375, 2.370 (2s, 6H, H-6), 2.37-2.30 (m, 1H, H-2'β).
^{13}C{^{1}H} NMR (125.7 MHz, d_6-DMSO) δ 165.485, 165.319 (2C, C1); 144.062, 143.846 (2C, C5); 129.430, 129.316, 129.299, 129.247 (8C, C3,4); 126.705, 126.435 (2C, C2); 91.671 (C1'); 82.056 (C4'); 74.590 (C3'); 63.974 (C5'); 37.832 (C2'); 21.188, 21.166 (2C, C6).
HRMS (ESI +): calcd. for $C_{21}H_{21}N_3NaO_5^+$ [M+Na]$^+$ 418.1379; found 418.1374.

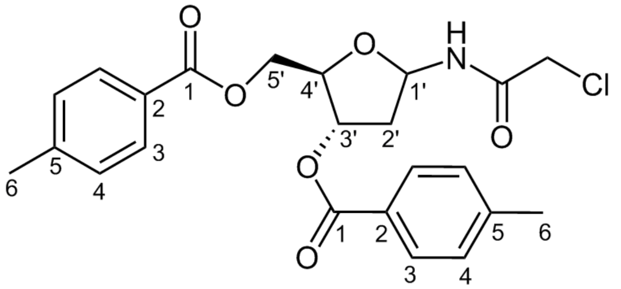

2-Deoxy-3,5-bis[*O*-(4-methylbenzoyl)]-α,β-D-*erythro*-pentofuranosyl 2-chloroacetamide (**16**) was obtained by a two-step one-pot procedure starting from 2-deoxyribofuranosyl azide **15**.
To a solution of azide **15** (19.8 g, 50.0 mmol) in CH_2Cl_2 (250 mL), Pd/C (2.5 g, 10% Pd) was added, and the mixture was vigorously stirred 3 h at room temperature under H_2. After the reduction of azide was complete (control by TLC in CH_2Cl_2), the mixture was filtered, cooled to 0 ºC and Et_3N (7.65 mL, 55 mmol), followed by chloroacetyl chloride (4.38 mL, 55 mmol), were added. Then, the reaction mixture was stirred on a melting ice bath overnight. After filtering, the organic layer was washed with H_2O (300 mL), sat. $NaHCO_3$ (300 mL), dried with Na_2SO_4, filtered and evaporated in vacuo. Purification was performed by column chromatography on silica gel (20–45 µm particles size, 40 × 150 mm column), eluting with a gradient of acetone (0 → 10%) in CH_2Cl_2 over 30 min at a flow rate of 100 mL/min. Fractions containing product were evaporated in vacuo, and resulting solid was dissolved in AcOEt (50 mL) upon heating and precipitated with hexane (250 mL) overnight at −20 ºC. Solids were filtered, washed with hexane/AcOEt (8:2, v/v) and dried in vacuo to result in chloroacetamide **16** (8.56 g, 38%) with β/α ratio about 1:1.

R_f 0.53 (β-**16**), 0.50 (α-**16**) (AcOEt/hexane, 4:6, v/v).
Pure β-**16** can be obtained by crystallisation of anomeric mixture from toluene.

β-**16**:
^{1}H NMR (500 MHz, d_6-DMSO) δ 9.01 (d, 1H, $^{3}J_{NH,1'}$ = 8.9 Hz), 7.89 (m, 4H, H-3), 7.33 (m, 4H, H-4), 5.84-5.77 (m, 1H, H-1'), 5.50 (m, 1H, H-3'), 4.40 (m, 2H, H-5'), 4.31 (m, 1H, H-4'), 4.11 (s, 2H, C*H*$_2$Cl); 2.384, 2.378 (2s, 6H, H-6); 2.36 (m, 2H, H-2').
^{13}C{^{1}H} NMR (125.8 MHz, d_6-DMSO) δ 166.4 (*C*ONH), 165.5, 165.3 (2C, C1), 144.0, 143.8 (2C, C5), 129.4, 129.35, 129.3 (8C, C3,4), 126.7, 126.6 (2C, C2), 80.3 (C4'), 80.1 (C1'), 75.0 (C3'), 64.5 (C5'), 42.6 (*C*H$_2$Cl), 35.9 (C2'), 21.18, 21.17 (2C, C6).

α-**16** (NMR data extracted from 75/25 mixture of α/β-**16**):
^{1}H NMR (400 MHz, d_6-DMSO) δ 8.77 (d, 1H, $^{3}J_{NH,1'}$ = 7.9 Hz), 7.89 (m, 4H, H-3), 7.33 (m, 4H, H-4), 5.83-5.75 (m, 1H, H-1'), 5.41 (m, 1H, H-3'), 4.50 (m, 1H, H-4'), 4.41 (m, 2H, H-5'), 4.11 (d, 2H, *J* = 1.4 Hz, C*H*$_2$Cl); 2.76 (m, 1H, H-2'), 2.39, 2.38 (2s, 6H, H-6); 2.14 (dt, 1H, *J* = 14.2 Hz, *J* = 3.8 Hz, H-2').
HRMS (ESI +): calcd. for $C_{23}H_{24}{}^{35}ClNNaO_6^+$ [M+Na]$^+$ 468.1190; found 468.1188.

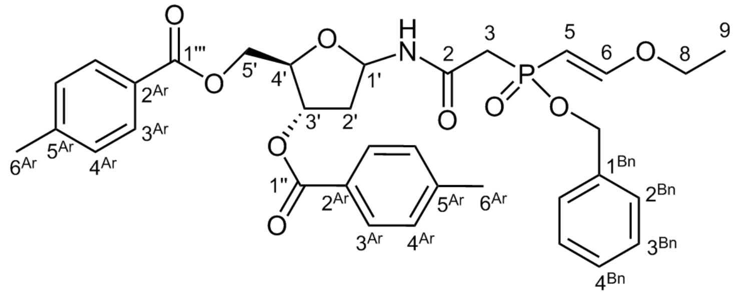

N-{2-Deoxy-3,5-bis[*O*-(4-methylbenzoyl)]-α,β-D-*erythro*-pentofuranosyl}-2-[(benzyloxy)((*E*)-2-ethoxyvinyl)phosphoryl]acetamide (**17**).
To a solution of chloroacetamide **16** (18.2 g, 40.9 mmol) in dichloroethane (80 mL), phosphinate **10** (13.9 g, 61.3 mmol) followed by HMDS (34.0 mL, 0.16 mol) were added and the reaction mixture was stirred 24 h at 90 ºC (oil bath). The hot mixture was carefully quenched with MeOH (15 mL, *caution*: exothermic reaction), evaporated in vacuo and purified by column chromatography on silica gel eluting with a stepwise gradient of acetone (0→40%) in toluene. Fractions containing both anomers of **17** were combined, evaporated and dried in vacuo resulting in linear **17** (8.36 g, 32%) as a mixture of two anomers.
Analytical sample of **17** (0.50 g) was subjected to the separation of anomers by column chromatography on silica gel (20–45 µm particles size, 25 × 150 mm column) eluting with a gradient of acetone (0→30%) in CH_2Cl_2 over 20 min at a flow rate of 40 mL/min. Fractions containing pure anomers were combined, evaporated and dried in vacuo.

α-**17**:
R_f 0.42 (acetone/CH_2Cl_2, 15:85, v/v).
^{1}H NMR (700 MHz, d_6-DMSO) δ 8.630, 8.627 (2d, 1H, $^{3}J_{NH,1'}$ = 8.3 Hz, N*H*), 7.94, 7.86 (2m, 4H, H-3^{Ar}), 7.40-7.24 (m, 9H, H-2^{Bn},3^{Bn},4^{Bn},4^{Ar}), 7.09 (m, 1H, H-6), 5.78 (m, 1H, H-1'), 5.39 (m, 1H, H-3'), 5.02-4.88 (m, 3H, H-5, C*H*$_2$Ph), 4.47-4.36 (m, 3H, H-4',5'), 3.86 (m, 2H, H-8), 3.06-2.90 (m, 2H, H-3), 2.75 (m, 1H, H-2'β), 2.38, 2.374, 2.370 (3s, 6H, H-6^{Ar}), 2.03, 1.98 (2dt, 1H, *J* = 14.3 Hz, *J* = 3.9 Hz, H-2'α), 1.19 (m, 3H, H-9).
^{13}C{^{1}H} NMR (176.1 MHz, d_6-DMSO) δ 165.01, 164.98 (C2); 162.55, 162.51 (2d, $^{2}J_{C6,P}$ = 17.9 Hz, C6); 165.52, 165.50, 165.49 (2C, C1'',1'''); 143.89, 143.87, 143.81 (2C, C5^{Ar}); 137.04 (m, *C*H$_2$Ph); 129.60, 129.59, 129.33, 129.25, 129.18, 129.17, 128.27, 128.25, 127.81, 127.79, 127.37, 127.33 (13C, C3^{Ar},4^{Ar},2^{Bn},3^{Bn},4^{Bn}); 126.65, 126.57 (2C, C2^{Ar}); 91.39, 91.35 (2d, $^{1}J_{C5,P}$ = 140.0 Hz, C5); 80.13, 80.11 (C4'); 80.07, 80.04 (C1'); 75.01, 74.97 (C3'); 65.83 (C8); 64.77 (d, $^{2}J_{C,P}$ = 5.6 Hz,

CH$_2$Ph); 64.35 (C5'); 39.13, 39.11 (2d, $^1J_{C3,P}$ = 93.5 Hz, C3); 36.89, 36.87 (C2'), 21.18, 21.16 (2C, C6^{Ar}); 14.149, 14.140 (C9).
^{31}P{^{1}H} NMR (202.5 MHz, d_6-DMSO, extn. ref. 85% H_3PO_4) δ 39.44, 38.32 (diastereomers).
HRMS (ESI +): calcd. for $C_{34}H_{38}NNaO_9P^+$ $[M+Na]^+$ 658.2182; found 658.2176.

β-**17**:
Rf 0.30 (acetone/CH_2Cl_2, 15:85, v/v).
^{1}H NMR (700 MHz, d_6-DMSO) δ 8.84-8.78 (m, 1H, NH), 7.91-7.87 (m, 4H, H-3^{Ar}), 7.40-7.26 (m, 9H, H-4^{Ar},2^{Bn},3^{Bn},4^{Bn}); 7.10, 7.09 (2dd, 1H, J = 13.7 Hz, J = 11.1 Hz, H-6); 5.82-5.76 (m, 1H, H-1'), 5.51-5.45 (m, 1H, H-3'), 5.01-4.90 (m, 3H, H-5,CH_2Ph), 4.43-4.33 (m, 2H, H-5'), 4.31-4.26 (m, 1H, H-4'), 3.92-3.84 (m, 2H, H-8), 3.02-2.88 (m, 2H, H-3); 2.385, 2.372, 2.368 (3s, 6H, H-6^{Ar}); 2.35-2.17 (m, 2H, H-2'), 1.215, 1.206 (2t, 3H, J = 7.0 Hz, H-9).
^{13}C{^{1}H} NMR (176.1 MHz, d_6-DMSO) δ 165.53 (br, C1'''); 165.28-165.18 (2C, C2,1''); 162.54 (d, $^2J_{C,P}$ = 17.8 Hz), 162.45 (d, $^2J_{C,P}$ = 17.5 Hz) (C6); 143.955, 143.946, 143.78, 143.76 (2C, C5^{Ar}); 137.08 (d, $^3J_{C,P}$ = 7.1 Hz), 137.03 (d, $^3J_{C,P}$ = 6.8 Hz) (C1^{Bn}); 129.39, 129.34, 129.30 (br), 129.28, 129.26, 128.27, 128.26, 127.81, 127.80, 127.42, 127.34 (13C, C3^{Ar},4^{Ar},2^{Bn},3^{Bn},4^{Bn}); 126.68, 126.59 (2C, C2^{Ar}); 91.41, 91.29 (2d, $^1J_{C,P}$ = 141.3 Hz, C5); 80.07 (br, C4'); 79.80, 79.77 (C1'); 75.08, 75.04 (C3'); 65.78 (br, C8); 64.79 (d, $^2J_{C,P}$ = 5.6 Hz, CH$_2$Ph); 64.59, 64.58 (C5'); 39.39, 39.34 (2d, $^1J_{C,P}$ = 94 Hz, C3, from DEPT); 36.1 (C2'); 21.18, 21.16 (2C, C6^{Ar}); 14.16, 14.15 (C9).
^{31}P{^{1}H} NMR (202.5 MHz, d_6-DMSO, extn. ref. 85% H_3PO_4) δ 39.1, 38.9 (diastereomers).
HRMS (ESI +): calcd. for $C_{34}H_{38}NNaO_9P^+$ $[M+Na]^+$ 658.2182; found 658.2177.

Anomerically pure **17** can be obtained starting from anomerically pure **16**, no isomerisation happens during the reaction.

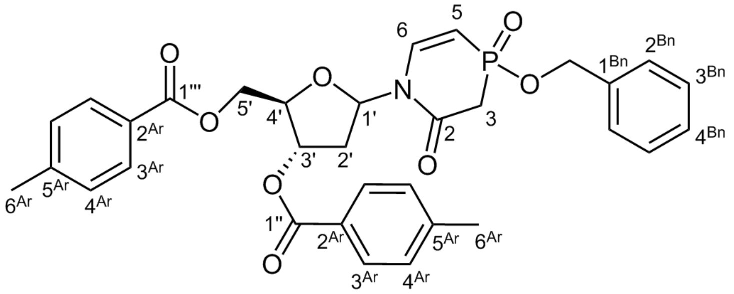

4-(Benzyloxy)-1-{2-deoxy-3,5-bis[O-(4-methylbenzoyl)]-α,β-D-*erythro*-pentofuranosyl}-3-hydro-1,4-azaphosphinin-2(1H)-one-4-oxide (**14**). Linear nucleoside **17** (11.4 g, 17.5 mmol) was co-evaporated with CH_3CN (100 mL), dissolved in CH_3CN (110 mL) and TMSOTf (634 µL, 3.51 mmol) was added. The mixture was stirred 2.5 h at 40 ºC (oil bath), quenched with Et_3N (1.20 mL), evaporated to dryness in vacuo and purified by column chromatography on silica gel (40 × 150 mm column) eluting with a gradient of acetone (0→15%) in CH_2Cl_2 over 20 min at a flow rate of 100 mL/min. Fractions containing cyclic **14** were combined and evaporated in vacuo resulting in 6.65 g (64 %) of yellowish foam. The α/β ratio determined from ^{31}P NMR is about 3:2.
R_f 0.56-0.46 (acetone/CH_2Cl_2, 15:85, v/v). Visually consists of three separate spots close to each other; UV-active, stains with $KMnO_4$.

^{1}H and ^{13}C NMR (d_6-DMSO) are not very informative because compound is present as a mixture of two anomers, α and β, both having two diastereomers on phosphorus atom.
^{1}H NMR (700 MHz, d_6-DMSO) δ 7.93-7.81 (m, 4H, H-3^{Ar}), 7.52-7.20 (m, 10H, H-4^{Ar},2^{Bn},3^{Bn},4^{Bn},6), 6.40-6.16 (m, 1H, H-1'), 5.57-5.46 (m, 1H, H-5).
^{31}P{^{1}H} NMR (202.5 MHz, d_6-DMSO, extn. ref. 85% H_3PO_4) δ 31.47, 31.24 (α-anomer), 30.71, 30.39 (β-anomer).
^{1}H-^{1}H COSY clearly shows four cross-spots of H-5 and H-6.
HRMS (ESI +): calcd. for $C_{32}H_{32}NNaO_8P^+$ $[M+Na]^+$ 612.1763; found 612.1763.

The cyclisation of pure α-**17** and β-**17** leads to **14** with the same α/β ratio ≈3:2.

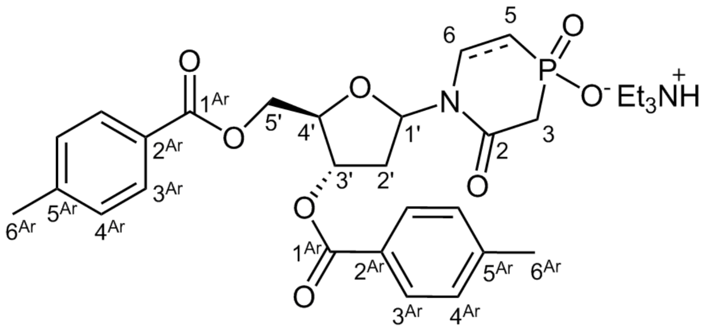

General procedure for preparation of 1-{2-deoxy-3,5-bis[*O*-(4-methylbenzoyl)]-α,β-D-*erythro*-pentofuranosyl}-4-hydroxy-3-hydro-1,4-azaphosphinin-2(1*H*),4-dione (**18**) and 1-{2-deoxy-3,5-bis[*O*-(4-methylbenzoyl)]-α,β-D-*erythro*-pentofuranosyl}-4-hydroxy-1,4-azaphosphinan-2,4-dione (**24**), triethylammonium salts.

To a solution of benzyl-protected nucleoside **14** (1.00 g, 1.70 mmol) in CH_2Cl_2 (17 mL), Et_3N (0.47 mL, 3.38 mmol) followed by the catalyst (0.50 g of 5%Pd/$CaCO_3$/3%Pb for the synthesis of **18** or 0.50 g of 10%Pd/C for the synthesis of **24**) were added and the reaction mixture was stirred 1.5 h (for **18**) or overnight (for **24**) under H_2 at room temperature. The catalyst was filtered off, the filtrate was evaporated, dried in vacuo and stock solution in H_2O (10 mL) was prepared with concentration of **18** (**24**) of ≈ 100 mg/mL. Individual anomers (α and β) were separated by column chromatography on 21 × 129 mm column packed in-lab with Jupiter C18 HPLC bulk media, 15 µm. A 3 mL portion of stock solution of **18**(**24**) was diluted to 15 mL with H_2O, loaded on the column and eluted with the following gradient of CH_3CN in H_2O at flow rate of 10 mL/min: 0→13% over 4 min, then 13→28% over 31 min. α-**18** (α-**24**) Elutes as a first significant peak followed by β-**18** (β-**24**) anomer.

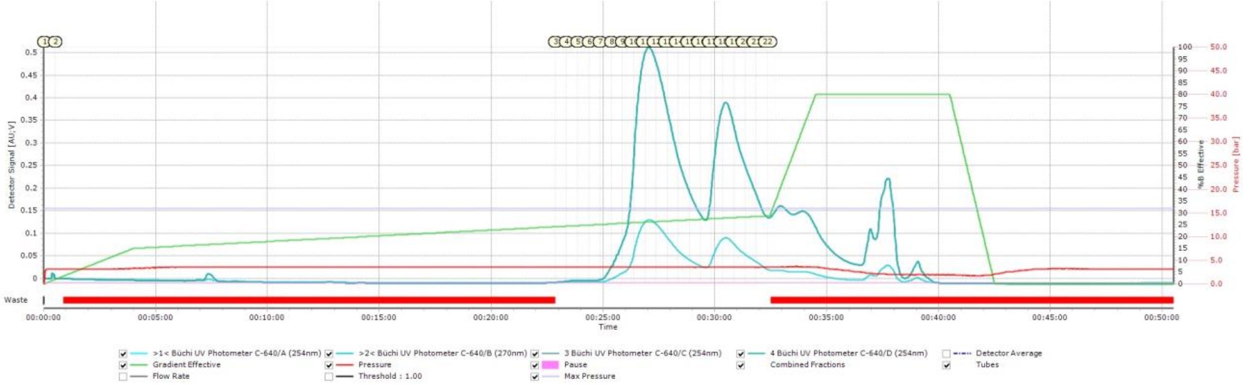

Figure S1. Chromatogram showing separation of compound **18**.

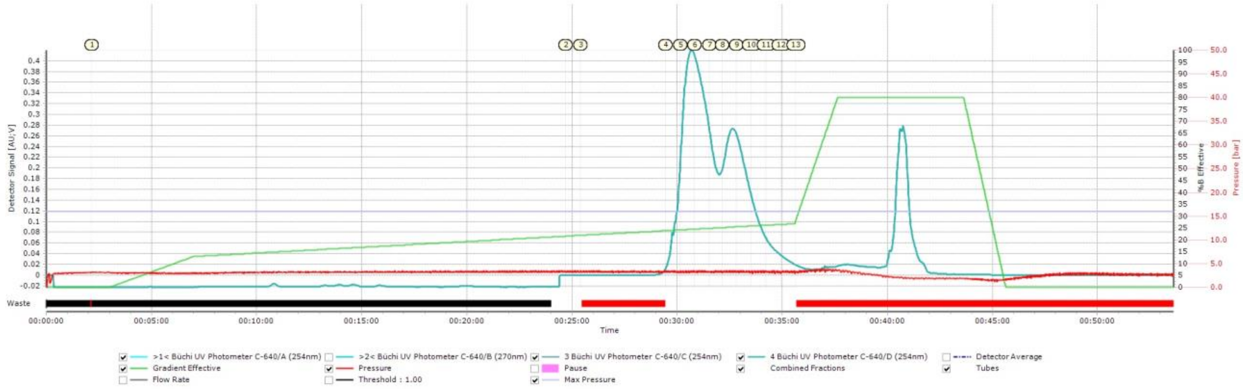

Figure S2. Chromatogram showing separation of compound **24**.

Fractions containing pure α- and β-anomers were combined separately, all volatile solvents were evaporated in vacuo and the residue was freeze-dried from H_2O.

α-**18**, triethylammonium salt:
Yield: 346 mg (34%)
R_f 0.47 ($MeOH/Et_3N/CH_2Cl_2$, 10:5:85, v/v/v)
^{1}H NMR (700 MHz, d_6-DMSO) δ 7.89 (m, 4H, H-3^{Ar}), 7.34 (m, 4H, H-4^{Ar}), 6.96 (dd, 1H, $^3J_{H6,P}$ = 34.6 Hz, $^3J_{H5,H6}$ = 10.9 Hz, H-6), 6.33 (dd, 1H, J = 7.6 Hz, J = 4.0 Hz, H-1'), 5.495 (dt, 1H, J = 7.0 Hz, J = 2.3 Hz, H-3'), 5.44 (dd, 1H, $^3J_{H5,H6}$ = 10.9 Hz, $^2J_{H5,P}$ = 7.3 Hz, H-5), 4.74 (dt, 1H, J = 4.8 Hz, J = 2.1 Hz, H-4'), 4.42 (dd, 1H, J = 11.7 Hz, J = 5.3 Hz, H-5'), 4.385 (dd, 1H, J = 11.7 Hz, J = 4.6 Hz, H-5'), 2.97 (q, 6H, J = 7.3 Hz, NCH_2CH_3), 2.87 (m, 1H, H-2'β), 2.81-2.62 (m, 2H, H-3), 2.38 (s, 6H, H-6^{Ar}), 2.09 (m, 1H, H-2'α), 1.14 (t, 9H, J = 7.3 Hz, NCH_2CH_3).
^{13}C{^{1}H} NMR (176.1 MHz, d_6-DMSO) δ 168.3 (C2, from HMBC); 165.46, 165.19 (2C, C1^{Ar}); 144.02, 143.89 (2C, C5^{Ar}); 133.30 (br, C6); 129.46, 129.39, 129.35, 129.28 (8C, C3^{Ar},4^{Ar}); 126.58, 126.40 (2C, C2^{Ar}); 109.5 (C5, from HSQC), 84.38 (C1'), 82.26 (C4'), 74.79 (C3'), 64.29 (C5'), 45.0 (3C, NCH_2CH_3), 41.3 (C3, from HSQC), 36.77 (C2'); 21.19, 21.18 (2C, C6^{Ar}), 8.4 (3C, NCH_2CH_3).
^{31}P{^{1}H} NMR (202.5 MHz, d_6-DMSO, extn. ref. 85% H_3PO_4) δ 7.17 (br).
HRMS (ESI -): calcd. for $C_{25}H_{25}NO_8P^-$ $[M-Et_3NH]^-$ 498.1323; found 498.1358.

β-**18**, triethylammonium salt:
Yield: 210 mg (21%)
R_f 0.47 ($MeOH/Et_3N/CH_2Cl_2$, 10:5:85, v/v/v)
^{1}H NMR (700 MHz, d_6-DMSO) δ 7.90 (m, 4H, H-3^{Ar}), 7.35 (m, 4H, H-4^{Ar}), 6.65 (dd, 1H, $^3J_{H6,P}$ = 33.0 Hz, $^3J_{H5,H6}$ = 10.8 Hz, H-6), 6.37 (dd, 1H, J = 8.4 Hz, J = 6.4 Hz, H-1'), 5.51 (m, 1H, H-3'), 5.26 (dd, 1H, $^3J_{H5,H6}$ = 10.8 Hz, $^2J_{H5,P}$ = 7.1 Hz, H-5), 4.58 (dd, 1H, J = 11.8 Hz, J = 3.9 Hz, H-5'), 4.50 (dd, 1H, J = 11.8 Hz, J = 4.6 Hz, H-5'), 4.355 (m, 1H, H-4'), 2.9 (m, 6H, NCH_2CH_3), 2.68-2.51 (m, 2H, H-3), 2.39 (s, 6H, H-6^{Ar}), 2.35-2.26 (m, 2H, H-2'), 1.12 (t, 9H, J = 7.3 Hz, NCH_2CH_3).
^{13}C{^{1}H} NMR (176.1 MHz, d_6-DMSO) δ 169.21 (d, $^2J_{C2,P}$ = 6.7 Hz, C2); 165.49, 165.28 (2C, C1^{Ar}); 144.02, 143.93 (2C, C5^{Ar}); 131.13 (br, C6); 129.43, 129.40, 129.33, 129.28 (8C, C3^{Ar},4^{Ar}); 126.57, 126.50 (2C, C2^{Ar}); 112.54 (d, $^1J_{C5,P}$ = 121.3 Hz, C5), 82.72 (C1'), 80.395 (C4'), 74.70 (C3'), 64.12 (C5'), 44.8 (3C, NCH_2CH_3), 42.11 (d, $^1J_{C3,P}$ = 77.2 Hz, C3); 35.20 (C2'), 21.20, 21.18 (2C, C6^{Ar}), 8.5 (3C, NCH_2CH_3).
^{31}P{^{1}H} NMR (202.5 MHz, d_6-DMSO, extn. ref. 85% H_3PO_4) δ 7.50 (br).
HRMS (ESI -): calcd. for $C_{25}H_{25}NO_8P^-$ $[M-Et_3NH]^-$ 498.1323; found 498.1356.

α-**24**, triethylammonium salt:
Yield: 235 mg (23%)
R_f 0.38 ($MeOH/Et_3N/CH_2Cl_2$, 10:5:85, v/v/v)
^{1}H NMR (700 MHz, d_6-DMSO) δ 7.88 (m, 4H, H-3^{Ar}), 7.35 (m, 4H, H-4^{Ar}), 6.37 (dd, 1H, J = 7.8 Hz, J = 5.5 Hz, H-1'), 5.46 (m, 1H, H-3'), 4.59 (m, 1H, H-4'), 4.39 (dd, 1H, J = 11.7 Hz, J = 5.2 Hz, H-5'), 4.35 (dd, 1H, J = 11.7 Hz, J = 4.5 Hz, H-5'), 3.53 (m, 2H, H-6), 3.37 (q, 6H, J = 7.2 Hz, NCH_2CH_3), 2.75 (m, 1H, H-2'), 2.52-2.30 (m, 2H, H-3), 2.39, 2.38 (2s, 6H, H-6^{Ar}), 1.97 (m, 1H, H-2'), 1.46 (m, 2H, H-5), 1.23 (t, 9H, J = 7.2 Hz, NCH_2CH_3).
HRMS (ESI -): calcd. for $C_{25}H_{27}NO_8P^-$ $[M-Et_3NH]^-$ 500.1480; found 500.1511.

β-**24**, triethylammonium salt:
Yield: 197 mg (19%)
R_f 0.38 ($MeOH/Et_3N/CH_2Cl_2$, 10:5:85, v/v/v)
^{1}H NMR (700 MHz, d_6-DMSO) δ 7.89 (m, 4H, H-3^{Ar}), 7.35 (m, 4H, H-4^{Ar}), 6.33 (dd, 1H, J = 9.2 Hz, J = 5.8 Hz, H-1'), 5.47 (m, 1H, H-3'), 4.57 (dd, 1H, J = 11.8 Hz, J = 4.0 Hz, H-5'), 4.44 (dd, 1H, J = 11.8 Hz, J = 4.5 Hz, H-5'), 4.26 (m, 1H, H-4'), 3.37 (q, 6H, J = 7.2 Hz, NCH_2CH_3), 3.31 (m, 2H, H-

6), 2.43, 2.34 (2m, 2H, H-3), 2.24 (m, 2H, H-2'), 2.39 (s, 6H, H-6^{Ar}), 1.30 (m, 2H, H-5), 1.23 (t, 9H, *J* = 7.2 Hz, NCH_2CH_3).
HRMS (ESI -): calcd. for $C_{25}H_{27}NO_8P^-$ $[M-Et_3NH]^-$ 500.1480; found 500.1512.

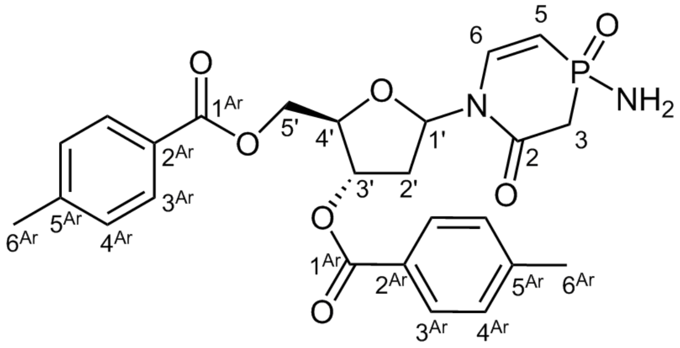

4-Amino-1-{2-deoxy-3,5-bis[*O*-(4-methylbenzoyl)]-α,β-ᴅ-*erythro*-pentofuranosyl}-3-hydro-1,4-azaphosphinin-2(1*H*),4-dione (**19**) was prepared similar to the previously described protocol [8]. To a solution of phosphinic salt **18** (mixture of anomers, 0.71 g, 1.18 mmol) in $CHCl_3$ (24 mL), oxalyl chloride (0.20 mL, 2.37 mmol) was added and the mixture was stirred 15 min at room temperature (gas evolution). Satd. NH_3 solution in $CHCl_3$ (5 mL) was added in one portion and the mixture was stirred 10 min, evaporated and purified by column chromatography (25 × 150 mm column) on silica gel eluting with a gradient of MeOH (0→8%) in CH_2Cl_2 over 15 min at a flow rate of 40 mL/min. Fractions containing the product were evaporated in vacuo resulting in **19** as a yellowish solid (0.27 g, 46%).

R_f 0.35 ($MeOH/CH_2Cl_2$, 7:93, v/v)
^{1}H NMR (500 MHz, d_6-DMSO) δ 7.93-7.87 (m, 4H, H-3^{Ar}); 7.37-7.32 (m, 4H, H-4^{Ar}); 7.24 (t, *J* = 11.0), 7.16 (t, *J* = 11.1), 7.08 (dd, *J* = 10.9, *J* = 5.2), 7.01 (dd, *J* = 11.0, *J* = 5.3) (1H, H-6); 6.34 (m), 6.27 (m) (1H, H-1'); 5.57-5.49 (m, 1H, H-3'); 5.48-5.40 (m), 5.39-5.33 (m) (1H, H-5); 4.86-4.78 (m), 4.41 (m) (1H, H-4'); 4.46-4.39 (m, 2H, H-5'); 3.16-2.93 (m, 2H, H-3); 2.93-2.86 (m), 2.42-2.34 (m), 2.39 (s, 6H, H-6^{Ar}); 2.23-2.12 (m) (2H, H-2').
^{13}C{^{1}H} NMR (125.8 MHz, d_6-DMSO) δ 166.63, 166.58 (C2); 165.49, 165.48, 165.45, 165.25, 165.21, 165.17 (2C, C1^{Ar}); 144.04, 144.02, 143.99, 143.96, 143.92, 143.91 (2C, C5^{Ar}); 136.87, 136.71, 136.49, 136.45 (C6); 129.65-129.20 (8C, C3^{Ar},4^{Ar}); 126.67, 126.57, 126.55, 126.53, 126.52, 126.46, 126.42, 126.33 (2C, C2^{Ar}); 104.49 (d, $^1J_{C5,P}$ = 115.9 Hz), 104.38 (d, $^1J_{C5,P}$ = 116.3 Hz), 103.68 (d, $^1J_{C5,P}$ = 116.5 Hz), 103.43 (d, $^1J_{C5,P}$ = 116.2 Hz) (C5); 85.52, 84.77, 83.42, 82.95 (C1'); 83.04, 82.57, 80.76, 80.55 (C4'); 74.76, 74.65, 74.58, 74.41 (C3'); 64.24, 64.19, 64.17, 64.07 (C5'); 40.75 (d, $^1J_{C3,P}$ = 74.4 Hz), 40.63 (d, $^1J_{C3,P}$ = 74.8 Hz), 40.50 (d, $^1J_{C3,P}$ = 74.0 Hz), 40.42 (d, $^1J_{C3,P}$ = 74.2 Hz) (C3, from DEPT); 37.45, 36.97, 35.53, 35.40 (C2'); 21.20, 21.18 (2C, C6^{Ar}).
^{31}P{^{1}H} NMR (202.5 MHz, d_6-DMSO, extn. ref. 85% H_3PO_4) δ 16.3, 15.9.
HRMS (ESI +): calcd. for $C_{25}H_{27}N_2NaO_7P^+$ $[M+Na]^+$ 521.1453; found 521.1452.

Starting from isomerically pure α-**18**, only α-**19** was obtained indicating that no significant anomerisation happened during the reaction.

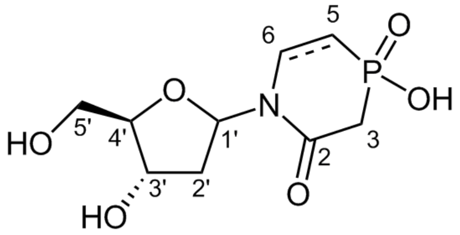

General procedure for preparation of 1-(2-deoxy-α,β-D-*erythro*-pentofuranosyl)-4-hydroxy-3-hydro-1,4-azaphosphinin-2(1*H*),4-dione (**Va**) or 1-(2-deoxy-α,β-D-*erythro*-pentofuranosyl)-4-hydroxy-1,4-azaphosphinan-2,4-dione (**Vc**).

Toluoyl-protected nucleoside **18** or **24** in a form of triethylammonium salt (0.60 g, 1.00 mmol) was dissolved in 28% aq. NH_3 (50 mL) and stirred overnight at room temperature. Silica gel (2 g) was added to the mixture, all volatiles were removed in vacuo, and nucleoside pre-absorbed on silica was dried in vacuo. The nucleoside was dry-loaded on a column with silica gel (12 × 150 mm) and purified eluting with a gradient of H_2O (0→50%) in CH_3CN over 25 min at a flow rate of 10 mL/min. Fractions containing a nucleoside were evaporated in vacuo to remove CH_3CN and then freeze-dried from H_2O.

Compound α-**Va** was prepared starting from compound α-**18**. Yield: 206 mg (78%).

R_f 0.15 (H_2O/CH_3CN, 20:80, v/v), UV-active

^{1}H NMR (700 MHz, 20 mM Na-phosphate, pH 7.0, 10% v D_2O, intn. ref. acetone) δ 7.14 (dd, 1H, $^3J_{H6,P}$ = 36.3 Hz, $^3J_{H5,H6}$ = 11.1 Hz, H-6), 6.24 (dd, 1H, J = 7.5 Hz, J = 5.3 Hz, H-1'), 5.67 (dd, 1H, $^3J_{H5,H6}$ = 11.1 Hz, $^2J_{H5,P}$ = 7.6 Hz, H-5), 4.38 (m, 1H, H-3'), 4.20 (m, 1H, H-4'), 3.70 (dd, 1H, J = 12.4 Hz, J = 3.5 Hz, H-5'), 3.60 (dd, 1H, J = 12.4 Hz, J = 5.6 Hz, H-5'), 3.05-2.87 (m, 2H, H-3), 2.65 (m, 1H, H-2'β), 2.00 (dt, 1H, J = 14.4 Hz, J = 4.7 Hz, H-2'α).

$^{13}C\{^1H\}$ NMR (176.0 MHz, 20 mM Na-phosphate, pH 7.0, 10% v D_2O, intn. ref. acetone) δ 170.79 (d, $^2J_{C2,P}$ = 5.1 Hz, C2), 136.39 (C6), 107.86 (d, $^1J_{C5,P}$ = 124.2 Hz, C5), 87.60 (C4'), 85.52 (C1'), 71.07 (C3'), 62.00 (C5'), 40.13 (d, $^1J_{C3,P}$ = 81.5 Hz, C3), 39.16 (C2').

$^{31}P\{^1H\}$ NMR (202.5 MHz, 20 mM Na-phosphate, pH 7.0, 10% v D_2O, extn. ref. 85% H_3PO_4) δ 18.04.

HRMS (ESI -): calcd. for $C_9H_{13}NO_6P^-$ $[M-H]^-$ 262.0486; found 262.0502.

ε_{258} 4230 L· mol^{-1}·cm^{-1}

Compound β-**Va** was prepared starting from compound β-**18**. Yield: 243 mg (92%).

R_f 0.15 (H_2O/CH_3CN, 20:80, v/v), UV-active

^{1}H NMR (700 MHz, 20 mM Na-phosphate, pH 7.0, 10% v D_2O, intn. ref. acetone) δ 6.97 (dd, 1H, $^3J_{H6,P}$ = 36.0 Hz, $^3J_{H5,H6}$ = 11.0 Hz, H-6), 6.37 (t, 1H, J = 7.1 Hz, H-1'), 5.68 (dd, 1H, $^3J_{H5,H6}$ = 11.0 Hz, $^2J_{H5,P}$ = 7.6 Hz, H-5), 4.38 (m, 1H, H-3'), 3.92 (m, 1H, H-4'), 3.78 (dd, 1H, J = 12.3 Hz, J = 3.7 Hz, H-5'), 3.70 (dd, 1H, J = 12.3 Hz, J = 5.5 Hz, H-5'), 3.06-2.89 (m, 2H, H-3), 2.26-2.18 (m, 2H, H-2').

$^{13}C\{^1H\}$ NMR (176.0 MHz, 20 mM Na-phosphate, pH 7.0, 10% v D_2O, intn. ref. acetone) δ 170.86 (d, $^2J_{C2,P}$ = 5.2 Hz, C2), 135.93 (C6), 108.55 (d, $^1J_{C5,P}$ = 123.9 Hz, C5), 86.36 (C4'), 84.21 (C1'), 71.33 (C3'), 62.07 (C5'), 40.21 (d, $^1J_{C3,P}$ = 81.3 Hz, C3), 38.01 (C2').

$^{31}P\{^1H\}$ NMR (202.5 MHz, 20 mM Na-phosphate, pH 7.0, 10% v D_2O, extn. ref. 85% H_3PO_4) δ 17.65.

HRMS (ESI -): calcd. for $C_9H_{13}NO_6P^-$ $[M-H]^-$ 262.0486; found 262.0499.

Compound **Vc** as an anomeric mixture was prepared starting from compound **24**. Yield: 241 mg (39%).

R_f 0.13 (H_2O/CH_3CN, 25:75, v/v), non-UV-active, stains with 2% aq. H_2SO_4/heat.

The nucleoside exists as a pair of α and β anomers, therefore two sets of signals are present in NMR spectra. Designations “α” and “β” are used to distinguish between the isomers. Integral intensities of the signals are reported as fractional numbers that reflect the ratio of isomers.

^{1}H NMR (700 MHz, 20 mM Na-phosphate, pH 7.0, 10% v D_2O, internal. ref. d_4-TMSP) δ 6.33 (dd, 0.4H, J = 8.2 Hz, J = 6.5 Hz, H-1'-β), 6.21 (t, 0.6H, J = 6.7 Hz, H-1'-α), 4.37 (m, 1H, H-3'-α,β), 4.13 (m, 0.6H, H-4'-α), 3.89 (m, 0.4H, H-4'-β), 3.82-3.58 (m, 4H, H-6,5'-α,β), 2.94-2.78 (m, 2H, H-3-α,β), 2.59 (m, 0.6H, H-2'-α), 2.18 (ddd, 0.4H, J = 14.1 Hz, J = 8.3 Hz, J = 6.8 Hz, H-2'-β), 2.09 (ddd, 0.4H, J = 14.1 Hz, J = 6.4 Hz, J = 3.3 Hz, H-2'-β), 1.95-1.85 (m, 2.6H, H-2'-α,3-α,β).
^{13}C{^{1}H} NMR (176.0 MHz, 20 mM Na-phosphate, pH 7.0, 10% v D_2O, intn. ref. d_4-TMSP) δ 174.48 (d, $^{2}J_{C2,P}$ = 4.6 Hz, C2β), 174.24 (d, $^{2}J_{C2,P}$ = 4.8 Hz, C2α), 88.71 (C4'α), 88.09 (C4'β), 86.30 (C1'α), 85.81 (C1'β), 73.87 (C3'β), 73.17 (C3'α), 64.55 (C5'β), 64.16 (C5'α), 42.31 (d, $^{1}J_{C3,P}$ = 72.8 Hz, C3β), 42.26 (d, $^{1}J_{C3,P}$ = 72.9 Hz, C3α), 40.88 (d, $^{2}J_{C6,P}$ = 5.7 Hz, C6α), 40.54 (d, $^{2}J_{C6,P}$ = 5.7 Hz, C6β), 39.96 (C2'α), 38.71 (C2'β), 31.27 (br d, $^{1}J_{C5,P}$ = 96.1 Hz, C5α,β).
^{31}P{^{1}H} NMR (202.5 MHz, 20 mM Na-phosphate, pH 7.0, 10% v D_2O, extn. ref. 85% H_3PO_4) δ 36.51 (α), 36.20 (β).
HRMS (ESI -): calcd. for $C_9H_{13}NO_6P^-$ [M-H]$^-$ 264.0642; found 264.0658.

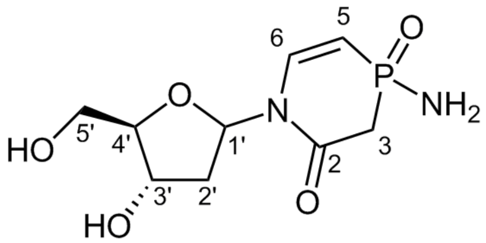

1-(2-Deoxy-α,β-ᴅ-*erythro*-pentofuranosyl)-4-amino-3-hydro-1,4-azaphosphinin-2(1*H*),4-dione (**Vb**). To a heterogeneous mixture of nucleoside **Va** (0.85 mmol) in $CHCl_3$ (50 mL), TBDPSCl (1.33 mL, 5.11 mmol) followed by Et_3N (1.18 mL, 8.48 mmol) were added and the mixture was refluxed for 2 h and evaporated in vacuo to dryness. The residue was dissolved in CH_2Cl_2 (20 mL) and oxalyl chloride (0.25 mL, 3.95 mmol) was added (slow gas evolution). The mixture was stirred 30 min at room temperature and quenched with satd. NH_3 solution in $CHCl_3$ (10 mL), evaporated in vacuo to dryness and purified by column chromatography (25 × 150 mm column) on silica gel eluting with a gradient of MeOH (0 → 10%) in CH_2Cl_2 over 20 min at a flow rate of 40 mL/min. Fractions containing UV-active product with R_f 0.16 (acetone/CH_2Cl_2, 1:1, v/v) were combined and evaporated to result in TBDPS-protected phosphinamide **20** (86 mg, 14%).

HRMS (ESI +): calcd. for $C_{41}H_{51}N_2NaO_5PSi_2^+$ [M+Na]$^+$ 761.2972; found 761.2968.

To a solution of compound **20** (68 mg, 92 µmol) in THF (5 mL), solution of $Bu_4NF \cdot 3H_2O$ (72 mg, 0.23 mmol) in THF (1 mL) was added and the mixture was stirred 1 h at room temperature. All volatiles were evaporated in vacuo and the residue was purified by column chromatography (12 × 150 mm column) on silica gel eluting with iPrOH over 20 min at a flow rate of 5 mL/min. Nucleoside **Vb** was eluted as a broad peak between 7–16 min:

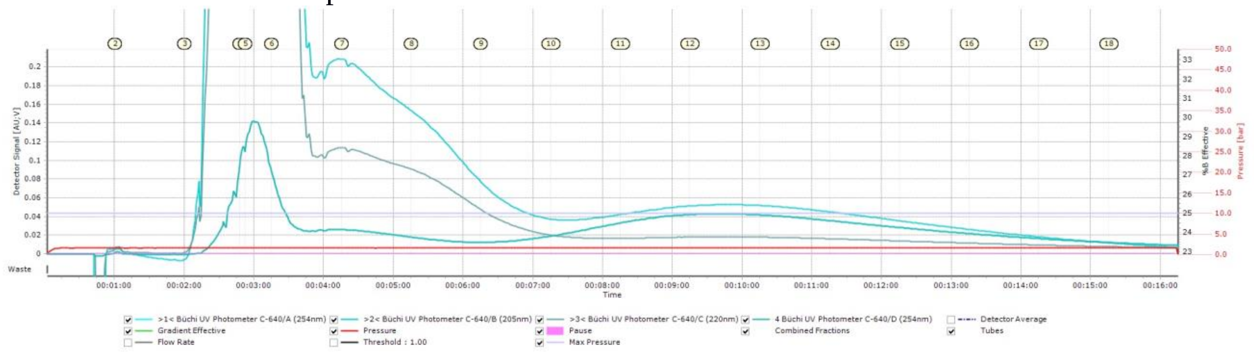

Figure S3. Chromatogram showing separation of compound **Vb** on a silica gel column.

Fractions containing compound **Vb** were evaporated in vacuo to dryness and the residue was purified by column chromatography on 21 × 129 mm column packed in-lab with Jupiter C18 HPLC bulk media, 15 μm, and eluting with H_2O at a flow rate of 10 mL/min. Phosphinamide nucleoside **Vb** elutes as a mixture of anomers between 5 and 6 min:

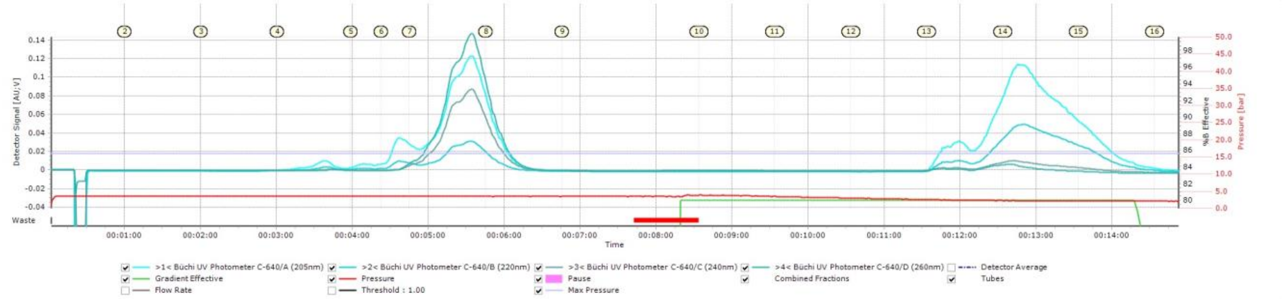

Figure S4. Chromatogram showing separation of compound **Vb** on a reverse-phase column.

Fractions containing the product were freeze-dried to result in 7.5 mg (31%) of **Vb** with α/β ratio ≈2:1.

R_f ≈0.5 (EtOH), broad spot, UV-active.

The nucleoside exists as a pair of α and β anomers, therefore two sets of signals are present in NMR spectra. Designations “α” and “β” are used to distinguish between the isomers. Integral intensities of the signals are reported mostly as fractional numbers that reflect the ratio of isomers.

^{1}H NMR (700 MHz, 20 mM Na-phosphate, pH 7.0, 10% v D_2O, intn. ref. acetone) δ 7.44 (dd, J = 38.5 Hz, J = 10.9 Hz), 7.42 (dd, J = 38.7 Hz, J = 10.9 Hz) (0.7H, H-6-α); 7.29 (dd, J = 38.4 Hz, J = 11.2 Hz), 7.26 (dd, J = 38.6 Hz, J = 11.0 Hz) (0.3H, H-6-β); 6.36, 6.35 (2t, 0.3H, J = 7.2 Hz, H-1'-β); 6.23 (dd, J = 7.7 Hz, J = 4.2 Hz), 6.22 (dd, J = 8.2 Hz, J = 4.2 Hz) (0.7H, H-1'-α); 5.73-5.62 (m, 1H, H-5-α,β); 4.39 (m, 1H, H-3'-α,β); 4.26 (m, 0.7H, H-4'-α); 3.95 (m, 0.3H, H-4'-β); 3.82-3.77, 3.74-3.69 (2m, 0.6H, H-5'-β); 3.69 (dd, J = 12.6 Hz, J = 3.9 Hz), 3.60 (dd, J = 12.6 Hz, J = 5.5 Hz) (1.7H, H-5'-α); 3.40-3.24 (m, 2H, H-3-α,β); 2.67 (m, 0.7H, H-2'-α); 2.28-2.16 (m, 0.6H, H-2'-β); 2.02, 2.00 (2t, 0.7H, J = 4.0 Hz, H-2'-α).

^{13}C{^{1}H} NMR (176.0 MHz, 20 mM Na-phosphate, pH 7.0, 10% v D_2O, intn. ref. acetone) δ 168.46, 168.44 (C2-β); 168.37, 168.34 (C2-α); 141.16, 141.10 (C6-α); 140.6 (C6-β); 101.28, 100.94 (2d, $^1J_{C5,P}$ = 120.0 Hz, C5-β); 100.25, 100.02 (2d, $^1J_{C5,P}$ = 120.0 Hz, C5-α); 88.35, 88.31 (C4'-α); 86.68, 86.66 (C4'-β); 86.49, 86.31 (C1'-α); 84.71, 84.66 (C1'-β); 71.32, 71.29 (C3'-β); 71.09 (C3'-α); 62.08, 62.03, 62.00 (C5'-α,β); 39.67, 39.54 (C2'-α); 39.62 (d, $^1J_{C3,P}$ = 76.9 Hz), 39.26 (d, $^1J_{C3,P}$ = 77.3 Hz) (C3-β); 39.56 (d, $^1J_{C3,P}$ = 76.9 Hz), 39.21 (d, $^1J_{C3,P}$ = 77.7 Hz) (C3-α); 38.43, 38.17 (C2'-β).

^{31}P{^{1}H} NMR (202.5 MHz, 20 mM Na-phosphate, pH 7.0, 10% v D_2O, extn. ref. 85% H_3PO_4) δ 26.34 (α), 25.89 (β).

HRMS (ESI +): calcd. for $C_9H_{15}N_2NaO_5P^+$ $[M+Na]^+$ 285.0616; found 285.0613.

UV-vis: ε_{262} 4730 L· mol^{-1} cm^{-1}

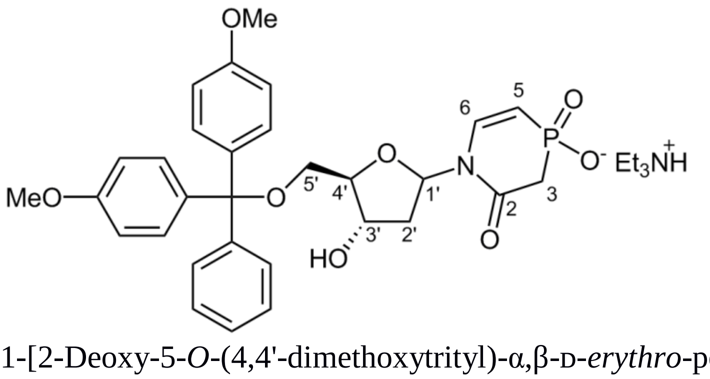

1-[2-Deoxy-5-*O*-(4,4'-dimethoxytrityl)-α,β-ᴅ-*erythro*-pentofuranosyl]-4-hydroxy-3-hydro-1,4-azaphosphinin-2(1*H*),4-dione, triethylammonium salt (**21**). To a solution of benzyl-protected nucleoside **14** (2.95 g, 5.00 mmol) in CH_2Cl_2 (50 mL), Et_3N (1.39 mL, 10.0 mmol) followed by 5%Pd/$CaCO_3$/3%Pb (1.48 g) were added and the reaction mixture was stirred 1.5 h under H_2 at room

temperature. The catalyst was filtered off, the filtrate was evaporated in vacuo and the residue was dissolved in 28% aq. NH_3 (150 mL) and stirred overnight at room temperature. The mixture was concentrated to the volume ≈50 mL and cooled at +4 °C for 1 h, crystals were filtered off and washed with a cold water. Combined water layer (200 mL) was extracted with AcOEt (200 mL), and water layer was evaporated in vacuo to dryness, co-evaporated with MeOH, CH_3CN and dried in vacuo resulting in compound **Va**.

Dry pyridine (200 mL) was added to compound **Va**, heating was required to dissolve major part of nucleoside **Va**. After the mixture was cooled down to room temperature, DMTCl (3.05 g, 9.00 mmol) was added in one portion and the reaction mixture was stirred for 24 h at room temperature. The residue obtained after evaporation of solvents in vacuo and co-evaporation with toluene was purified by column chromatography (25 × 150 mm column) on silica gel eluting with a gradient of solvent B (0→30%) in solvent A over 20 min at a flowrate 50 mL/min (solvent A: Et_3N/CH_2Cl_2 5:95 v/v, solvent B: Et_3N/MeOH 5:95 v/v). Fractions containing the product with R_f 0.4 ($MeOH/Et_3N/CH_2Cl_2$, 10:5:85, v/v/v) were combined and evaporated in vacuo resulting in 3.20 g (96%) of **21** as a mixture of anomers.

The stock solution of compound **21** in H_2O (32 mL) was prepared with concentration of **21** ≈100 mg/mL. Anomers α- and β- were separated by column chromatography on 21 × 129 mm column packed in-lab with Jupiter C18 HPLC bulk media, 15 µm. A 3-10 mL portions of stock solution of **21** were diluted to 15 mL with H_2O, loaded on the column and eluted with the following gradient of CH_3CN in H_2O at a flow rate of 10 mL/min: 0→10% over 4 min, then 10→45% over 31 min. β-**21** Elutes as a first significant peak followed by α-**21** anomer:

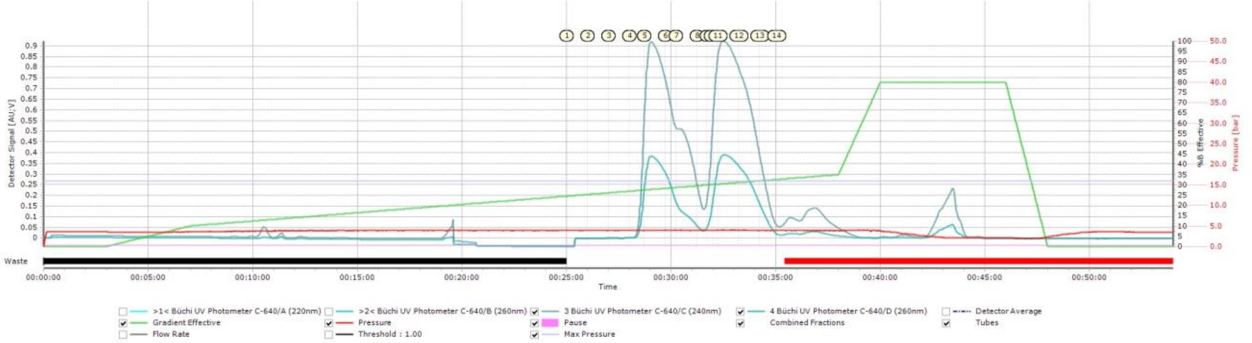

Figure S5. Chromatogram showing separation of α- and β-anomers of compound **21**.

Fractions containing pure α- and β-anomers were combined separately, Et_3N (1 mL) was added to each compound and all volatile solvents were evaporated in vacuo, the residue was co-evaporated with CH_3CN (2 × 200 mL) and CH_2Cl_2 (2 × 200 mL).

α-**21**, triethylammonium salt:
0.82 g (25%)
R_f 0.4 ($MeOH/Et_3N/CH_2Cl_2$, 10:5:85, v/v/v)
Contains $Et_3NCH_2Cl^+$ Cl^- as an impurity with a molar ratio α-23/impurity 1:0.6.
^{1}H NMR (700 MHz, d_6-DMSO) δ 5.35 (s, 2H, Et_3NCH_2Cl), 3.37 (q, 6H, J = 7.2 Hz, CH_3CH_2N), 1.23 (t, 9H, J = 7.2 Hz, CH_3CH_2N).
^{13}C{^{1}H} NMR (176.1 MHz, d_6-DMSO) δ 62.62 (Et_3NCH_2Cl), 52.00 (3C, CH_3CH_2N), 7.20 (3C, CH_3CH_2N).
^{1}H NMR (700 MHz, d_6-DMSO) δ 7.38 (m, 2H, H-2''), 7.31 (m, 2H, H-3''), 7.25 (m, 4H, H-2'''), 7.21 (m, 1H, H-4''), 6.89 (m, 4H, H-3'''), 6.86 (dd, 1H, $^3J_{H6,P}$ = 32.2 Hz, $J_{H5,H6}$ = 10.8 Hz, H-6), 6.33 (dd, 1H, J = 7.7 Hz, J = 5.7 Hz, H-1'), 5.38 (dd, 1H, $J_{H5,H6}$ = 10.8 Hz, $^2J_{H5,P}$ = 7.3 Hz, H-5), 4.08 (m, 1H, H-3'), 4.05 (m, 1H, H-4'), 3.73 (s, 6H, OCH_3), 3.02 (dd, 1H, J = 10.1 Hz, J = 3.3 Hz, H-5'), 2.90 (dd, 1H, J = 10.1 Hz, J = 5.2 Hz, H-5'), 2.54-2.38 (m, 9H, H-3,2',CH_3CH_2N), 1.69 (dt, 1H, J = 13.5 Hz, J = 4.9 Hz, H-2'), 0.93 (t, 9H, J = 7.1 Hz, CH_3CH_2N).

$^{13}C\{^1H\}$ NMR (176.1 MHz, d_6-DMSO) δ 169.66 (d, $^2J_{C6,P}$ = 5.4 Hz, C2), 158.03 (2C, C4'''), 144.87 (C1''); 135.63, 135.56 (2C, C1'''); 131.26 (C6), 129.66, 129.65 (4C, C2'''), 127.85 (2C, C3''), 127.67 (2C, C2''), 126.62 (C4''), 113.72 (d, $^1J_{C3,P}$ = 117.1 Hz, C5), 113.21 (4C, C3'''), 85.49 (*C*Ar$_3$), 85.19 (C4'), 82.95 (C1'), 70.98 (C3'), 64.30 (C5'), 55.02 (2C, O*C*H$_3$), 42.76 (d, $^1J_{C5,P}$ = 74.6 Hz, C3), 39.19 (C2', from DEPT).
$^{31}P\{^1H\}$ NMR (202.5 MHz, d_6-DMSO, extn. ref. 85% H_3PO_4) δ 5.66.
HRMS (ESI -): calcd. for $C_{30}H_{31}NO_8P^-$ [M-Et$_3$NH]$^-$ 564.1793; found 564.1829.

β-**21**, triethylammonium salt:
0.66 g (20%)
R_f 0.4 (MeOH/Et$_3$N/CH$_2$Cl$_2$, 10:5:85, v/v/v)
^1H NMR (700 MHz, d_6-DMSO) δ 7.39 (m, 2H, H-2''), 7.31 (m, 2H, H-3''), 7.26 (m, 4H, H-2'''), 7.21 (m, 1H, H-4''), 6.89 (m, 4H, H-3'''), 6.62 (dd, 1H, $^3J_{H6,P}$ = 32.0 Hz, $J_{H5,H6}$ = 10.8 Hz, H-6), 6.26 (t, 1H, J = 6.9 Hz, H-1'), 5.20 (dd, 1H, $J_{H5,H6}$ = 10.8 Hz, $^2J_{H5,P}$ = 7.3 Hz, H-5), 4.16 (m, 1H, H-3'), 3.77 (m, 1H, H-4'), 3.73 (s, 6H, OC*H$_3$*), 3.12 (m, 2H, H-5'), 2.53, 2.39 (2m, 2H, H-3), 1.91 (m, 2H, H-2').
$^{13}C\{^1H\}$ NMR (176.1 MHz, d_6-DMSO) δ 169.54 (d, $^2J_{C6,P}$ = 5.5 Hz, C2), 158.07 (2C, C4'''), 144.87 (C1''); 135.54, 135.45 (2C, C1'''); 130.30 (C6), 129.76, 129.74 (4C, C2'''), 127.85 (2C, C3''), 127.71 (2C, C2''), 126.67 (C4''), 113.89 (d, $^1J_{C3,P}$ = 117.0 Hz, C5); 113.23, 113.21 (4C, C3'''), 85.62 (*C*Ar$_3$), 84.41 (C4'), 82.29 (C1'), 70.45 (C3'), 63.79 (C5'), 55.05 (2C, O*C*H$_3$), 42.61 (d, $^1J_{C3,P}$ = 75.0 Hz, C3), 38.82 (C2').
$^{31}P\{^1H\}$ NMR (202.5 MHz, d_6-DMSO, extn. ref. 85% H_3PO_4) δ 5.59.
HRMS (ESI -): calcd. for $C_{30}H_{31}NO_8P^-$ [M-Et$_3$NH]$^-$ 564.1793; found 564.1828.

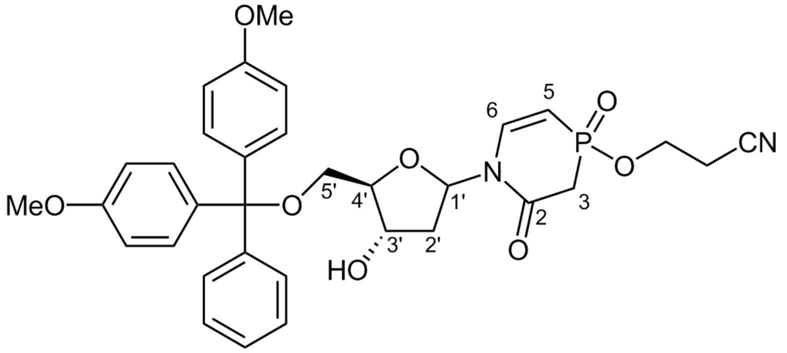

General procedure for preparation of 1-[2-Deoxy-5-*O*-(4,4'-dimethoxytrityl)-α,β-D-*erythro*-pentofuranosyl]-4-(2-cyanoethoxy)-3-hydro-1,4-azaphosphinin-2(1*H*),4-dione (**22**).
To a solution of pure α- or β-anomer of salt **21** (0.67 g, 1.00 mmol) in CH$_2$Cl$_2$ (20 mL), 3-hydroxypropionitrile (0.34 mL, 5.00 mmol), Et$_3$N (0.42 mL, 3.00 mmol), and TBTU (0.64 g, 2.00 mmol) were added, and the mixture was refluxed for 1 h. Reaction mixture was diluted with CH$_2$Cl$_2$ to 100 mL and washed with H$_2$O (100 mL). The emulsion formed was centrifuged for 5 min at 5,000 rpm and organic layer was separated and evaporated in vacuo to dryness. The residue was purified by column chromatography (21 × 129 mm column) on silica gel. Column was washed with Et$_3$N/CH$_2$Cl$_2$ (1:99, v/v, 200 mL) and CH$_2$Cl$_2$ (200 mL), sample was loaded on the column, and it was eluted with a gradient of iPrOH (0→15%) in CH$_2$Cl$_2$ over 20 min at a flow rate of 25 mL/min. The product was eluted as two separable diastereomers on phosphorus center, that were combined and evaporated in vacuo to dryness to result in α-**22** or β-**22** (both compounds appear as a yellowish foam).

α-**22**:
Yield: 165 mg (27%).
R_f 0.33, 0.30 (iPrOH/CH$_2$Cl$_2$, 7:93, v/v)
^1H NMR (500 MHz, d_6-DMSO) δ 7.60 (dd, J = 41.7 Hz, J = 11.3 Hz), 7.55 (dd, J = 41.1 Hz, J = 11.2 Hz), (1H, H-6); 7.37 (m, 2H, H-2''), 7.32 (m, 2H, H-3''), 7.27-7.20 (m, 5H, H-2''',4''), 6.90 (m, 4H, H-3'''); 6.30 (dd, J = 7.9 Hz, J = 3.9 Hz), 6.27 (dd, J = 7.9 Hz, J = 4.0 Hz) (1H, H-1'); 5.66-5.57 (m, 1H,

H-5), 4.23-4.17 (m, 1H, H-4'), 4.17-4.06 (m, 3H, H-3',C*H*$_2$CH$_2$CN), 3.74 (s, 6H, OC*H*$_3$); 3.62-3.22 (m, 2H, H-3), 3.08-3.00, 2.97-2.91 (2m, 2H, H-5'); 2.91-2.86 (m, 2H, CH$_2$C*H*$_2$CN), 2.62-2.50, 1.79-1.69 (2m, 2H, H-2').
^{13}C{^{1}H} NMR (125.7 MHz, d_6-DMSO) δ 165.66 (d, $^2J_{C2,P}$ = 5.3 Hz, C2), 158.07 (2C, C4'''); 144.77, 144.76 (C1''); 142.22, 142.13 (C6); 135.52, 135.51, 135.43, 135.40 (2C, C1'''); 129.65, 129.64 (4C, C2'''); 127.87 (2C, C3''), 127.63 (2C, C2''), 126.69 (C4''); 118.34, 118.31 (*C*N); 113.23 (4C, C3'''); 97.50 (d, $^1J_{C5,P}$ = 124.8 Hz), 97.25 (d, $^1J_{C5,P}$ = 125.2 Hz) (C5); 86.68, 86.67 (C4'); 85.66, 85.63 (*C*Ar$_3$); 84.63, 84.60 (C1'); 70.80, 70.78 (C3'); 64.05 (C5'); 59.48, 59.42 (d, ^2J = 5.4 Hz, *C*H$_2$CH$_2$CN); 55.03 (2C, O*C*H$_3$); 39.98, 39.72 (C2'); 36.92 (d, $^1J_{C3,P}$ = 83.5 Hz, C3), 19.42 (d, ^3J = 6.3 Hz, CH$_2$*C*H$_2$CN).
^{31}P{^{1}H} NMR (202.5 MHz, d_6-DMSO, extn. ref. 85% H_3PO_4) δ 32.9, 32.4.
HRMS (ESI +): calcd. For $C_{33}H_{35}N_2NaO_8P^+$ [M+Na]$^+$ 641.2029; found 641.2025.

β-**22**:
Yield: 450 mg (73%)
R_f 0.72, 0.69 (iPrOH/CH$_2$Cl$_2$, 10:90, v/v)
^{1}H NMR (500 MHz, d_6-DMSO) δ 7.38 (m, 2H, H-2''), 7.36-7.28 (m, 3H, H-6,3''), 7.28-7.21 (m, 5H, H-4'',2'''), 6.94-6.88 (m, 4H, H-3'''), 6.21 (m, 1H, H-1'), 5.34-5.25 (m, 2H, H-5,O*H*), 4.28-4.19 (m, 1H, H-3'), 4.17-4.05 (m, 2H, C*H*$_2$CH$_2$CN), 3.84 (m, 1H, H-4'), 3.76-3.73 (m, 6H, OC*H*$_3$), 3.50-3.29 (m, 2H, H-3), 3.25-3.12 (m, 2H, H-5'), 2.91-2.85 (m, 2H, CH$_2$C*H*$_2$CN), 2.11-1.95 (m, 2H, H-2').
^{13}C{^{1}H} NMR (125.7 MHz, d_6-DMSO) δ 165.56 (d, $^2J_{C6,P}$ = 5.7 Hz), 165.49 (d, $^2J_{C6,P}$ = 4.9 Hz) (C2); 158.13, 158.12, 158.11 (2C, C4'''); 144.72, 144.69 (C1''); 141.16, 140.97 (C6); 135.47, 135.41, 135.26, 135.24 (2C, C1'''); 129.75, 129.73 (4C, C2'''); 127.91 (2C, C3''), 127.69 (2C, C2''), 126.75 (C4''); 118.30, 118.26 (*C*N); 113.28, 113.25, 113.23 (4C, C3'''); 98.13 (d, $^1J_{C5,P}$ = 124.5 Hz), 97.47 (d, $^1J_{C5,P}$ = 125.6 Hz) (C5); 85.78, 85.76 (*C*Ar$_3$); 85.08 (C4'); 83.35, 83.25 (C1'); 70.27, 70.09 (C3'); 63.53, 63.41 (C5'); 59.61, 59.43 (d, ^2J = 5.5 Hz, *C*H$_2$CH$_2$CN); 55.06, 55.05 (2C, O*C*H$_3$); 39.45, 38.96 (C2'); 37.18 (d, $^1J_{C3,P}$ = 81.9 Hz), 36.86 (d, $^1J_{C3,P}$ = 83.7 Hz) (C3); 19.44, 19.38 (d, ^3J = 6.4 Hz, CH$_2$*C*H$_2$CN).
^{31}P{^{1}H} NMR (202.5 MHz, d_6-DMSO, extn. ref. 85% H_3PO_4) δ 32.2, 31.9.
HRMS (ESI +): calcd. for $C_{33}H_{35}N_2NaO_8P^+$ [M+Na]$^+$ 641.2029; found 641.2026.

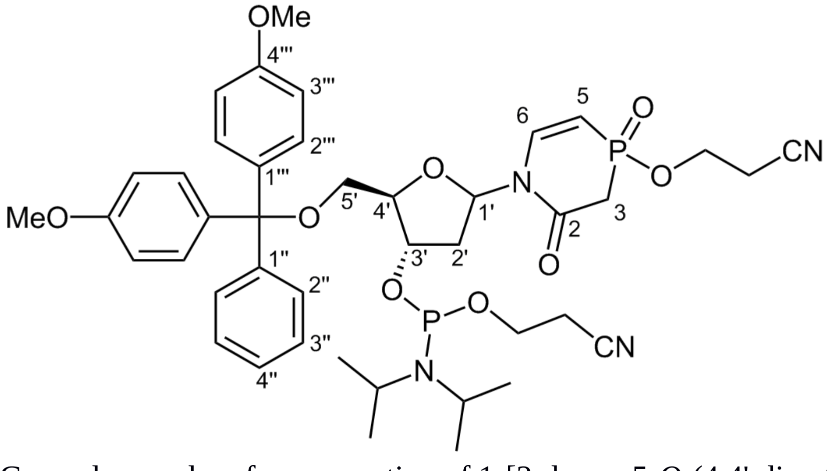

General procedure for preparation of 1-[2-deoxy-5-*O*-(4,4'-dimethoxytrityl)-3-*O*-(*N*,*N*-diisopropylamino-2-cyanoethoxyphosphanyl)-α,β-ᴅ-*erythro*-pentofuranosyl]-4-(2-cyanoethoxy)-3-hydro-1,4-azaphosphinin-2(1*H*),4-dione (**23**).
To a solution of pure α- or β-anomer of **22** (155 mg, 0.25 mmol) in dry CH$_2$Cl$_2$ (10 mL), Et$_3$N (209 µL, 1.50 mmol) followed by *N*,*N*-diisopropylamino-2-cyanoethoxychlorophosphine (145 mg, 0.63 mmol) were added and the mixture was stirred for 1 h at room temperature. Reaction mixture was diluted with CH$_2$Cl$_2$ to 100 mL, washed with satd. NaHCO$_3$ (100 mL), dried over Na$_2$SO$_4$, filtered and evaporated in vacuo to dryness. The residue was purified by column chromatography (12 × 150 mm column) on silica gel. Column was washed with Et$_3$N/CH$_2$Cl$_2$ (10:90, v/v, 100 mL) and CH$_2$Cl$_2$ (100 mL), sample was loaded on the column and it was eluted with a gradient of iPrOH (0→10%)

in CH_2Cl_2 over 10 min at a flow rate of 10 mL/min. Fractions containing product **23** were combined and evaporated in vacuo to dryness, the residue was co-evaporated with dry CH_2Cl_2 (2 × 100 mL) and finally freeze-dried from benzene.

α-**23**:
Yield: 91 mg (44%)
R_f ≈0.4 (iPrOH/CH_2Cl_2, 5:95, v/v), TLC plate treated with Et_3N/hexane (1:9, v/v) and dried prior to spotting.
^{1}H NMR (700 MHz, d_6-DMSO) δ 7.50-7.39 (m, 1H, H-6); 7.39-7.35 (m, 2H, H-2"); 7.34-7.29 (m, 2H, H-3"); 7.27-7.20 (m, 5H, H-2"',4"); 6.92-6.86 (m, 4H, H-3"'); 6.37-6.30 (m, 1H, H-1'); 5.63-5.53 (m, 1H, H-5); 4.44-4.28 (m, 2H, H-3',4'); 4.15-4.07, 3.73-3.61 (2m, 4H, C*H₂*CH₂CN); 3.737, 3.735 (2s, 6H, OC*H₃*); 3.55, 3.30 (2m, 2H, H-3); 3.55-3.48 (m, 2H, C*H*CH₃); 3.18-3.09, 3.02-2.95 (2m, 2H, H-5'); 2.92-2.85, 2.72-2.64 (2m, 4H, CH₂C*H₂*CN); 2.77-2.62, 2.04-1.81 (2m, 2H, H-2'); 1.15-1.01 (m, 12H, CHC*H₃*).
^{13}C{^{1}H} NMR (176.0 MHz, d_6-DMSO) δ 165.72-165.62 (C2); 158.12 (2C, C4"'); 144.64 (C1"); 141.60, 141.51, 141.47 (C6); 135.42, 135.36, 135.28, 135.26, 135.25, 135.23 (2C, C1"'); 129.62 (4C, C2"'); 127.88 (2C, C3"); 127.61, 127.57 (2C, C2"); 126.74 (C4"); 118.94, 118.84, 118.29, 118.21 (2C, CH₂CH₂*C*N); 113.23 (4C, C3"'); 97.88, 97.75 (2d, $^1J_{C5,P}$ = 125.3 Hz, C5); 85.84, 85.81, 85.80 (*C*Ar₃); 85.71, 85.69, 85.42, 85.39 (C4'); 84.82, 84.81, 84.78, 84.49 (C1'); 73.75, 73.65, 73.14, 73.06 (C3'); 63.7-63.6 (C5'); 59.58-59.40, 58.43, 58.39, 58.32, 58.28 (2C, *C*H₂CH₂CN); 55.03 (2C, O*C*H₃); 42.65, 42.57, 42.50 (2C, *C*HCH₃); 39.3, 39.0 (C2', from DEPT); 36.9 (br d, $^1J_{C3,P}$ = 83 Hz, C3); 24.34, 24.30, 24.28, 24.24, 24.19, 24.16 24.12 (CH*C*H₃); 19.86, 19.85, 19.82, 19.81, 19.77, 19.46, 19.45, 19.43, 19.41, 19.39 (2C, CH₂*C*H₂CN)
^{31}P{^{1}H} NMR (202.5 MHz, d_6-DMSO, extn. ref. 85% H_3PO_4) δ 149.00, 148.98, 148.52, 148.36 (P(III)); 32.50, 32.48, 32.09 (P(V)).
HRMS (ESI +): calcd. for $C_{42}H_{52}N_4NaO_9P_2^+$ $[M+Na]^+$ 841.3107; found 841.3103.

β-**23**:
Yield: 119 mg (58%)
R_f ≈0.4 (iPrOH/CH_2Cl_2, 5:95, v/v), TLC plate treated with Et_3N/hexane (1:9, v/v) and dried prior to spotting.
^{1}H NMR (700 MHz, d_6-DMSO) δ 7.41-7.36 (m, 2H, H-2"); 7.36-7.27 (m, 3H, H-6,3"); 7.27-7.22 (m, 5H, H-4",2"'); 6.93-6.87 (m, 4H, H-3"'); 6.22 (m, 1H, H-1'); 5.34 (m, 1H, H-5); 4.47 (m, 1H, H-3'); 4.10 (m, 2H, C*H₂*CH₂CN); 4.02-3.92 (m, 1H, H-4'); 3.76-3.57 (m, 2H, C*H₂*CH₂CN); 3.74 (m, 6H, OC*H₃*); 3.57-3.45 (m, 2H, C*H*CH₃); 3.26-3.19 (m, 2H, H-5'); 2.90-2.86 (m, 2H), 2.75 (m, 1H), 2.64 (m, 1H) (CH₂C*H₂*CN); 2.30-2.08 (m, 2H, H-2'); 1.14-0.96 (m, 12H, CHC*H₃*).
^{13}C{^{1}H} NMR (176.0 MHz, d_6-DMSO) δ 165.56 (m, C2); 158.16, 158.14 (2C, C4"'); 144.60 (C1"); 141.24, 141.05, 140.92, 140.76 (C6); 135.34, 135.28, 135.19, 135.14, 135.10 (2C, C1"'); 129.74, 129.73, 129.69 (4C, C2"'); 127.89 (2C, C3"); 127.69, 127.64 (2C, C2"); 126.78 (C4"); 118.93, 118.92, 118.75, 118.29, 118.28, 118.27, 118.22 (2C, CH₂CH₂*C*N); 113.24, 113.23, 113.22 (4C, C3"'); 99.0-97.2 (m, C5); 85.95, 85.92, 85.88, 85.85 (*C*Ar₃); 84.05, 84.03, 83.69, 83.66 (C4'); 83.40, 83.36, 83.21, 83.10 (C1'); 72.80 (d, $^2J_{C3',P}$ = 17.9 Hz), 72.58 (d, $^2J_{C3',P}$ = 17.6 Hz), 72.45 (d, $^2J_{C3',P}$ = 16.4 Hz), 72.20 (d, $^2J_{C3',P}$ = 16.3 Hz) (C3'); 63.06, 62.93, 62.90, 62.74 (C5'); 59.60, 59.59 (2d, ^2J = 5.4 Hz), 59.48 (d, ^2J = 5.7 Hz), 59.45 (d, ^2J = 5.9 Hz), 58.34, 58.33, 58.29 (3d, ^2J = 18.4 Hz) (2C, *C*H₂CH₂CN); 55.06, 55.05, 55.04 (2C, O*C*H₃); 42.62, 42.60, 42.55, 42.53 (2C, *C*HCH₃); 38.29, 38.18, 37.80, 37.65 (C2'); 37.5-36.4 (m, C3); 24.38-24.10 (4C, CH*C*H₃); 19.84-19.70, 19.47-19.31 (2C, CH₂*C*H₂CN).
^{31}P{^{1}H} NMR (202.5 MHz, d_6-DMSO, extn. ref. 85% H_3PO_4) δ 148.95, 148.90, 148.64, 148.56 (P(III)); 32.07, 32.01, 31.70, 31.69 (P(V)).
HRMS (ESI +): calcd. for $C_{42}H_{52}N_4NaO_9P_2^+$ $[M+Na]^+$ 841.3107; found 841.3102.

Determination of molar extinction coefficient of nucleosides Va and Vb

A sample of nucleoside (3–7 mg) was dissolved in a 20.0 mM phosphate buffer, containing 10 % D_2O at pH 7.0. ^{31}P NMR spectrum without ^{1}H decoupling was recorded overnight with a relaxation delay increased to 10 s. Concentration of nucleoside in the solution was determined by integration bands corresponding to phosphinate group of the nucleoside and phosphate buffer according to the equation:

$$c(nucleoside) = c(phosphate) \cdot \frac{I(nucleoside)}{I(phosphate)}$$

A dilution series with a factor 10 were prepared from the solution of nucleoside and UV absorption spectrum was recorded in the range 220–350 nm in 1 cm quartz cuvette. A molar extinction coefficient was determined using equation:

$$\varepsilon = \frac{OD}{c(nucleoside)}$$

A)

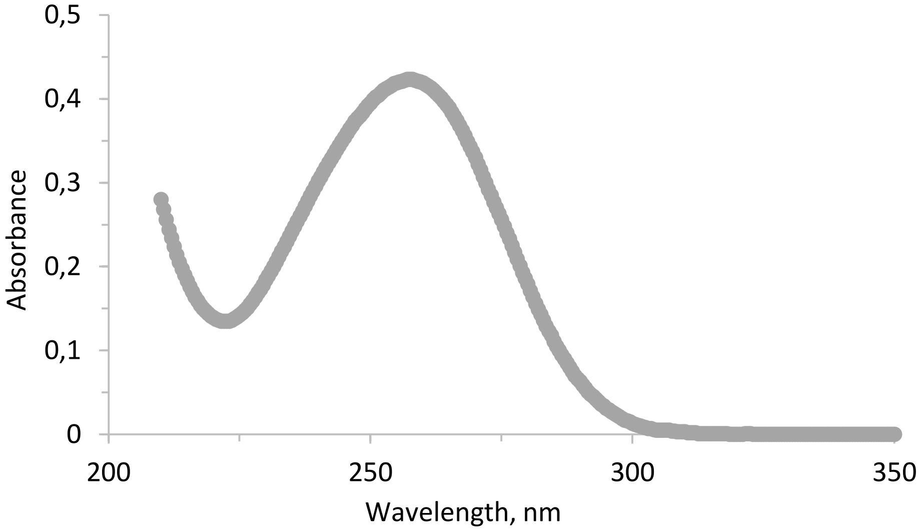

B)

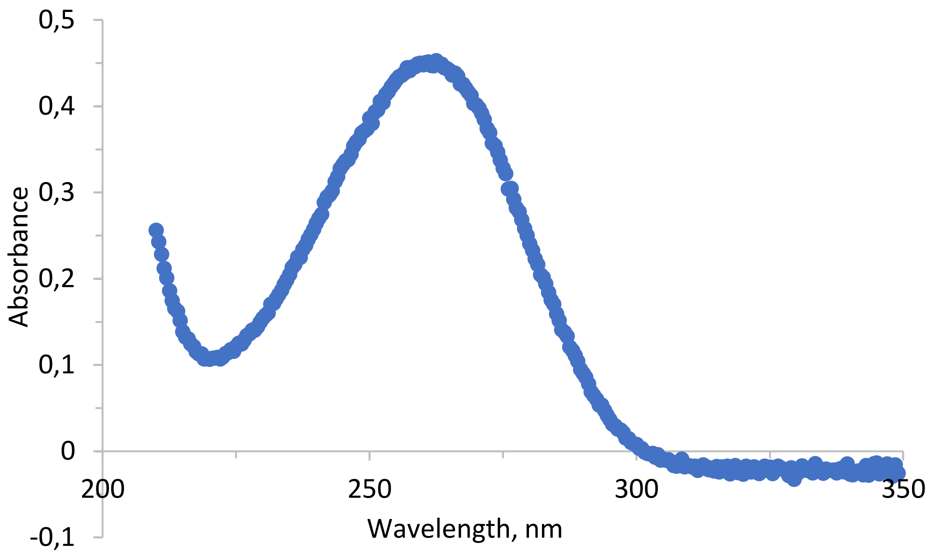

Figure S6. UV-vis spectra of compounds **Va** (A) and **Vb** (B) at 100 µM in 20 mM phosphate buffer, containing 10 % D_2O at pH 7.0.

Estimation of pK_a of nucleoside Va

A change of λ_{max} of nucleoside **Va** as a function of pH of the solution was plotted (Figure below) and pK_a was estimated to be ≤ 1.5.

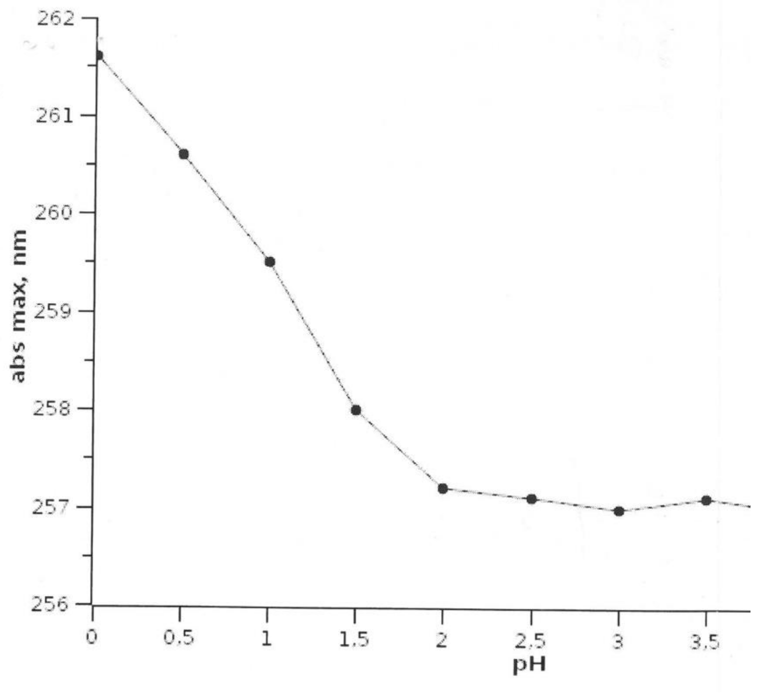

Figure S7. Change in λ_{max} of nucleoside **Va** as a function of pH.

Synthesis of modified oligonucleotides

Oligodeoxynucleotides were prepared on a MerMade-4 DNA/RNA synthesizer (BioAutomation) on a 5 µmol scale using standard manufacturer's protocol. Phosphoramidites α-**23** and β-**23** were used in the synthesis of modified oligonucleotide sequences. Each phosphoramidite (30 mg) was dissolved in 420 µL of freshly distilled, dry acetonitrile. Coupling time of modified nucleosides was increased to 5 min. After DNA assembly, the final detritylation step was performed and DMT-OFF oligonucleotides were cleaved from the solid support and deprotected with conc. ammonia solution (1.0 mL) at room temperature for 24 h. After filtering, ammonia was removed on a speed-vac (1h).

ONs were purified by HPLC using IE- column (TSKgel Super Q- 5PW). Buffer A (20 mM Tris-HCl, 1 mM Na_2-EDTA, pH 9.0), buffer B (20 mM Tris-HCl, 1 mM Na_2-EDTA, 1 M NaCl, pH 9.0). Gradients: 3.7 min 100 % A, convex curve gradient to 30 % B in 7.3 min, linear gradient to 50 % B in 18.5 min, concave gradient to 100 % B in 7.4 min, keep 100 % B for 7.4 min and then 100 % A in 7.4 min. Collected individual UV- absorbing fractions (λ=260 nm) were desalted using NAP- 25 column (GE Healthcare) against 'saltless buffer'. The composition of each fraction was confirmed by ESI- MS, and the fractions containing desired modified ONs were identified, analyzed by analytical RP-HPLC, ESI-MS (Figures S7 and S8) and concentrated by freeze-drying.

The oligonucleotides obtained were analysed by reverse-phase HPLC on 250/4.6 mm, 5 µm, 300 Å C18 column (Thermo Fisher Scientific) in a gradient of CH_3CN (0→20% for 20 min, 1.3 mL/min) in 0.1 M TEAA buffer (pH 7.0) with a detection at 260 nm ESI-MS was performed in negative mode using 10 µM aq. oligonucleotide solution supplemented with 20% MeOH.

A)

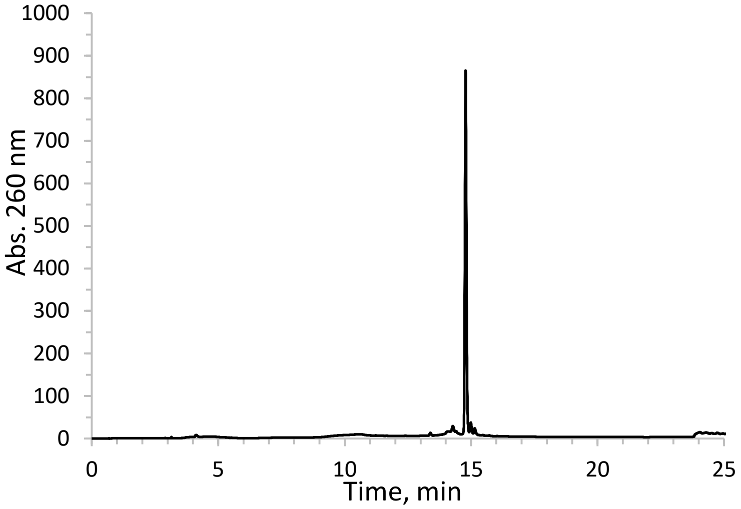

B)

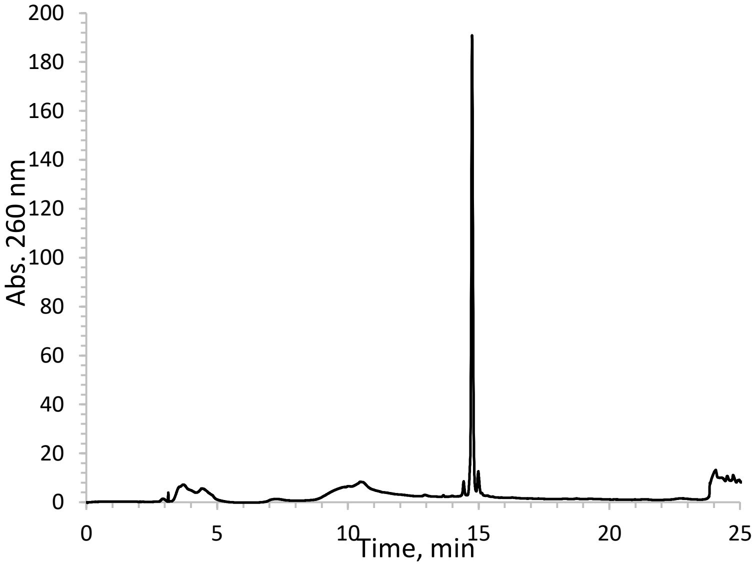

C)

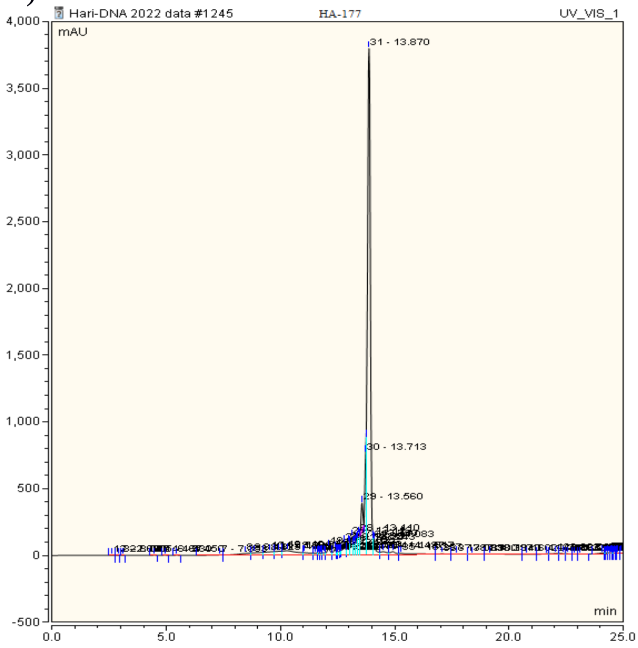

Figure S8. Analytical RP-HPLC profiles of oligonucleotides TTTT β-**Va** AT (A), TTTT α-**Va** AT (B) and TGCGCTT β-**Va** GCGCT (C). Note that peaks at 3–5, 9–11 and 24–26 min are artefacts form the column/buffer in A and B.

A)

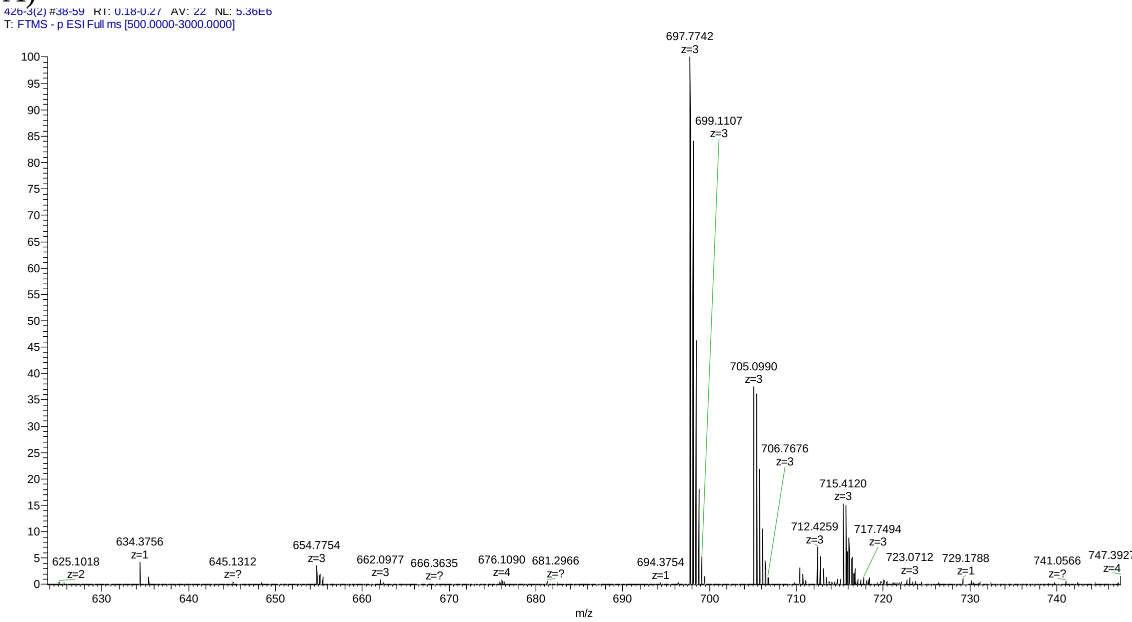
426-3(2) #38-59 RT: 0.18-0.27 AV: 22 NL: 5.36E6
T: FTMS - p ESI Full ms [500.0000-3000.0000]
697.7742 z=3
699.1107 z=3
705.0990 z=3
706.7676 z=3
715.4120 z=3
712.4259 z=3
717.7494 z=3
625.1018 z=2
634.3756 z=1
645.1312 z=?
654.7754 z=3
662.0977 z=3
666.3635 z=?
676.1090 z=4
681.2966 z=?
694.3754 z=1
723.0712 z=3
729.1788 z=1
741.0566 z=?
747.3927 z=4
m/z

B)

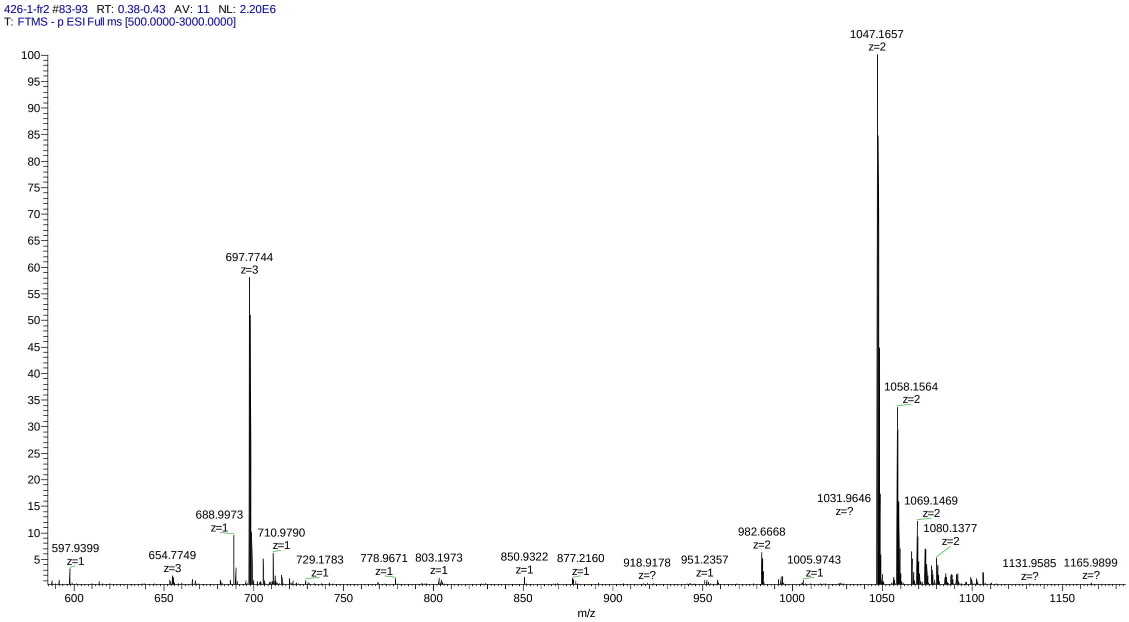
426-1-fr2 #83-93 RT: 0.38-0.43 AV: 11 NL: 2.20E6
T: FTMS - p ESI Full ms [500.0000-3000.0000]
1047.1657 z=2
697.7744 z=3
1058.1564 z=2
1031.9646 z=?
1069.1469 z=2
1080.1377 z=2
688.9973 z=1
710.9790 z=1
597.9399 z=1
654.7749 z=3
729.1783 z=1
778.9671 z=1
803.1973 z=1
850.9322 z=1
877.2160 z=1
918.9178 z=?
951.2357 z=1
982.6668 z=2
1005.9743 z=1
1131.9585 z=?
1165.9899 z=?
m/z

C)

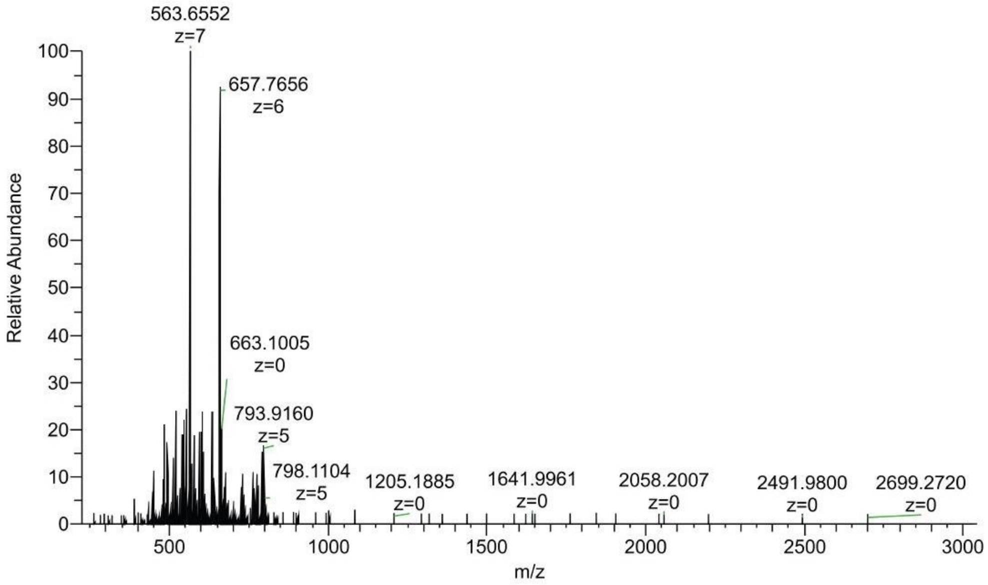

Figure S9. ESI-MS profiles of ODNs dTTTT β-**Va** AT (A), dTTTT α-**Va** AT (B) and TGCGCTT β-**Va** GCGCT (C). Calcd for [dTTTT **Va** AT -2H]$^{-2}$ = 1047.17 found 1047.17 for TTTT β-**Va** AT. Calcd for [dTTTT **Va** AT -3H]$^{-3}$ = 697.78 found 697.77 for both β and α ODN. Calcd for [TGCGCTT β-**Va** GCGCT – 7H]$^{-7}$ = 563.5193, found 563.6552.

Protein expression and purification

Recombinant human cytidine deaminase (hCDA) expressed in *E.Coli* was purchased form Sigma-Aldrich, product No SRP6372.
A3A mimic ($A3B_{CTD}$-QM-ΔL3-AL1swap) and wild-type A3A were expressed and purified as previously described.[9]

UV kinetic assay of hCDA-catalysed 2'-deoxycytidine deamination

UV spectroscopy was used to monitor hCDA-catalysed deamination of 2'-deoxycytidine at 286 nm in 100 mM NaCl, 50 mM phosphate buffer at 25 °C at various pH as mentioned in the text. Cary 300 Bio UV-vis spectrometer from Varian was used. Enzymatic reactions were performed in a quartz cuvette (0.1 cm). Deamination of 2'-deoxycytidine leads to a decrease in absorption at 286 nm (Figure S10).

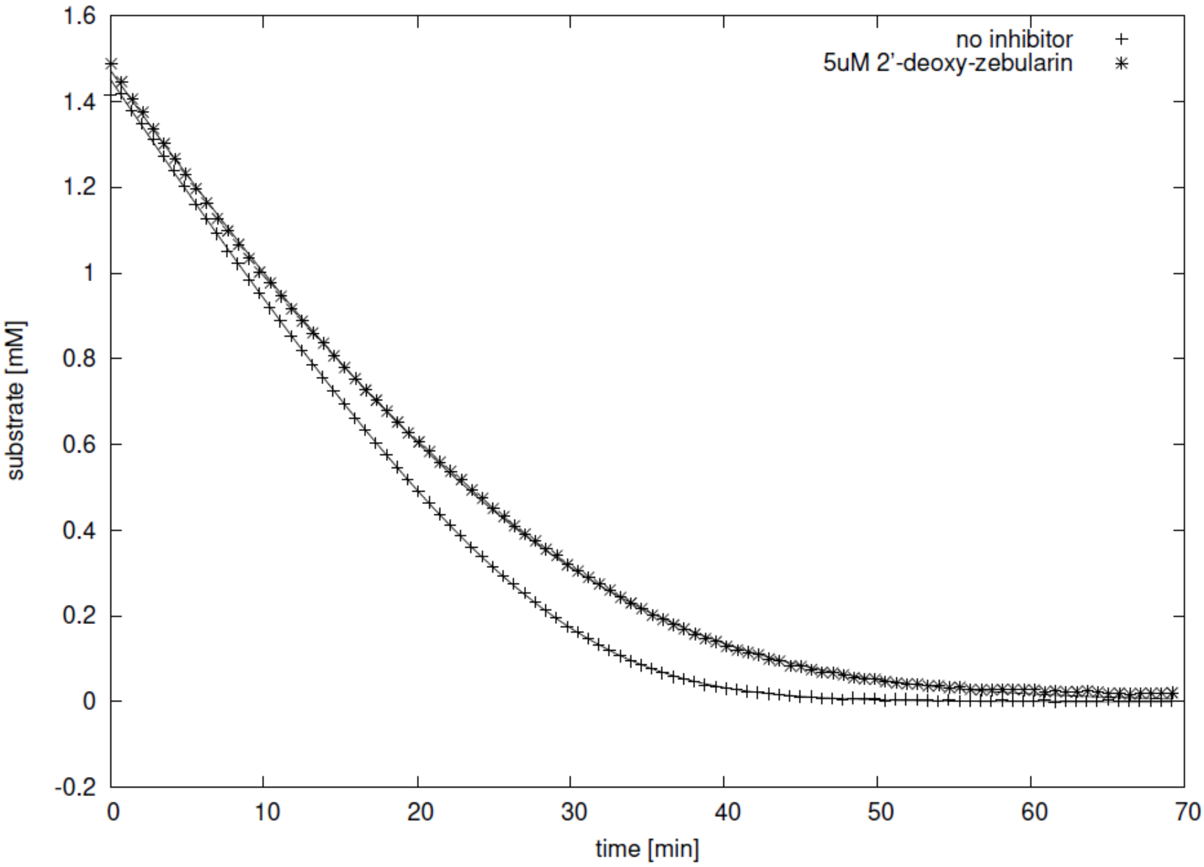

Figure S10. hCDA (27nM) catalysed deamination of 2'-deoxycitidine (~1.5 mM) in the absence (plus) and presence of 5 µM 2'-deoxyzebularine (**IIc**, asterisk) monitored by measuring absorbance at 286 nm over time in 100 mM NaCl, 1 mM TCEP, 50 mM phosphate buffer at 25 °C, at pH 7.4.

The uninhibited deamination (pluses, Figure S10) and the inhibited reaction start at about the same speed. As the reaction progresses, the inhibited reaction becomes slower, as its observed K_M is larger than in the uninhibited case. In order to be able to visualize the fitted curves, only every 5th data point is plotted. The data was then fitted using Gnuplot to the integrated Michaelis Menten equation [10]:

$$mm(t) = K_M * Lambert\ W\left(\frac{c}{K_M} * e^{\left(\frac{c}{K_M} - t * \frac{vmax}{K_M}\right)}\right) + d$$

where *mm(t)* calculates the current substrate concentration with respect to the time, *t*. The four parameters which are fitted are K_M and v_{max}, the Michaelis-Menten constants, *c* is the initial substrate concentration and *d* is a baseline correction parameter. A summary of the measured inhibition constants is given in Table 1 in the main text.

An increase in concentration of an inhibitor in enzymatic reaction leads to an increase in the observed K_M value. The plot of observed K_M values versus inhibitor concentration (Figures S11–S13) was then fitted with linear regression (y = ax + b) to derive the inhibition constant (K_i) using the following formula:

$$K_i = a/b$$

Uncertainty of K_i was calculated using error-propagation method.

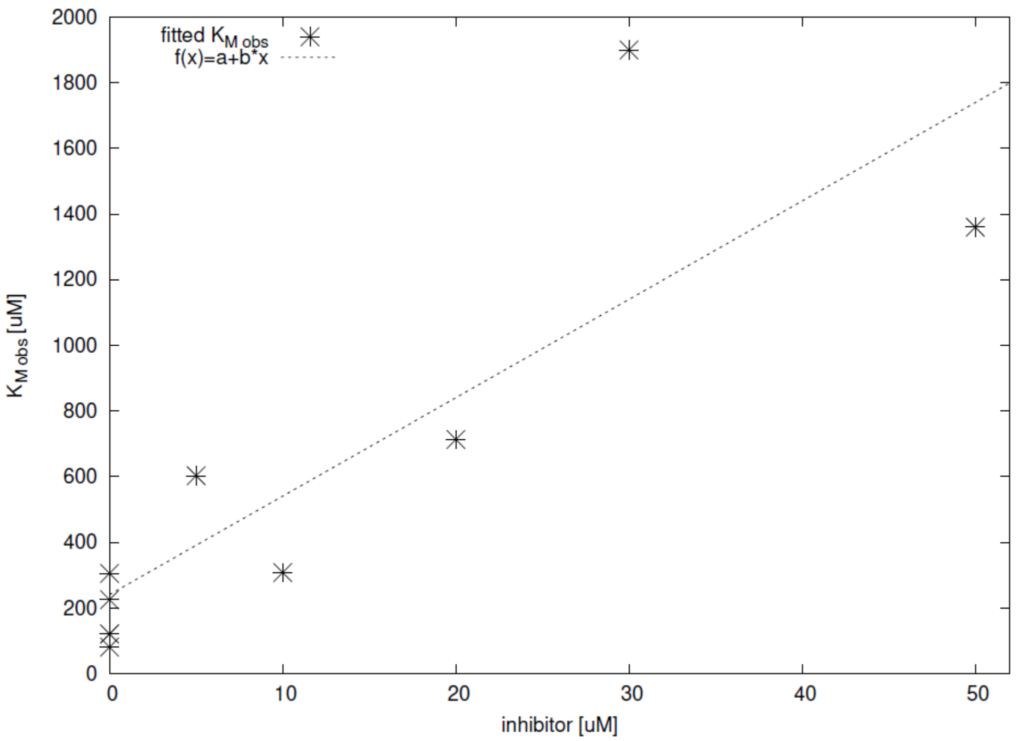

Figure S11. A plot of observed K_M versus various concentration of dZ (**IIc**) in hCDA (27 nM) catalysed deamination of 2'-deoxycitidine in 100 mM NaCl, 1 mM TCEP, 50 mM phosphate buffer at 25 °C, at pH 7.4.

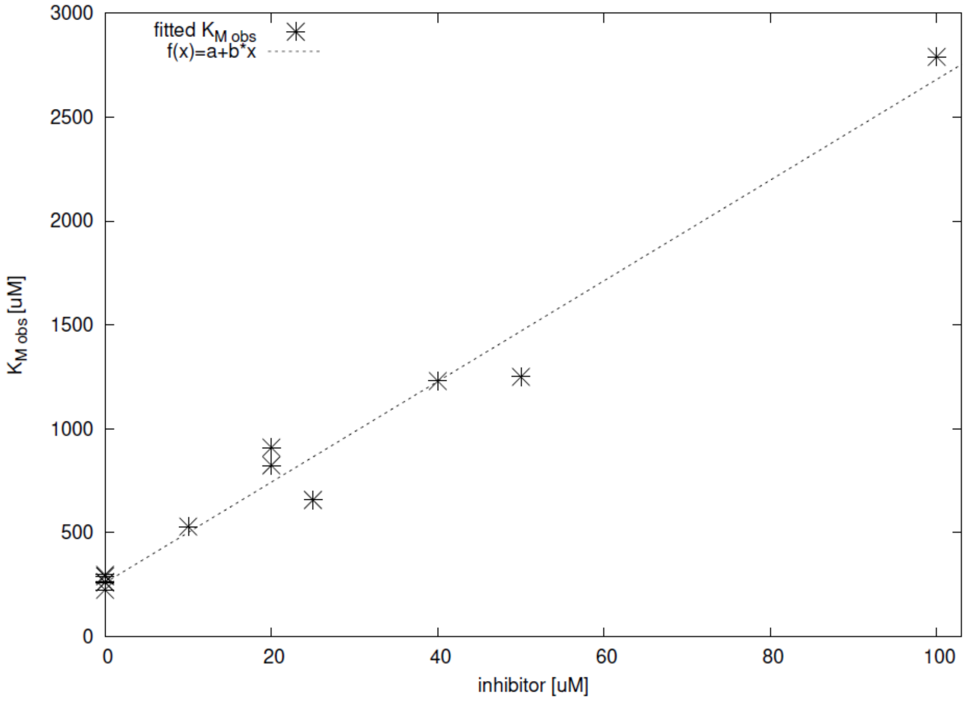

Figure S12. A plot of observed K_M versus concentration of compound **Vb** in hCDA (27 nM) catalysed deamination of 2'-deoxycitidine in 100 mM NaCl, 1 mM TCEP, 50 mM phosphate buffer at 25 °C, at pH 7.4.

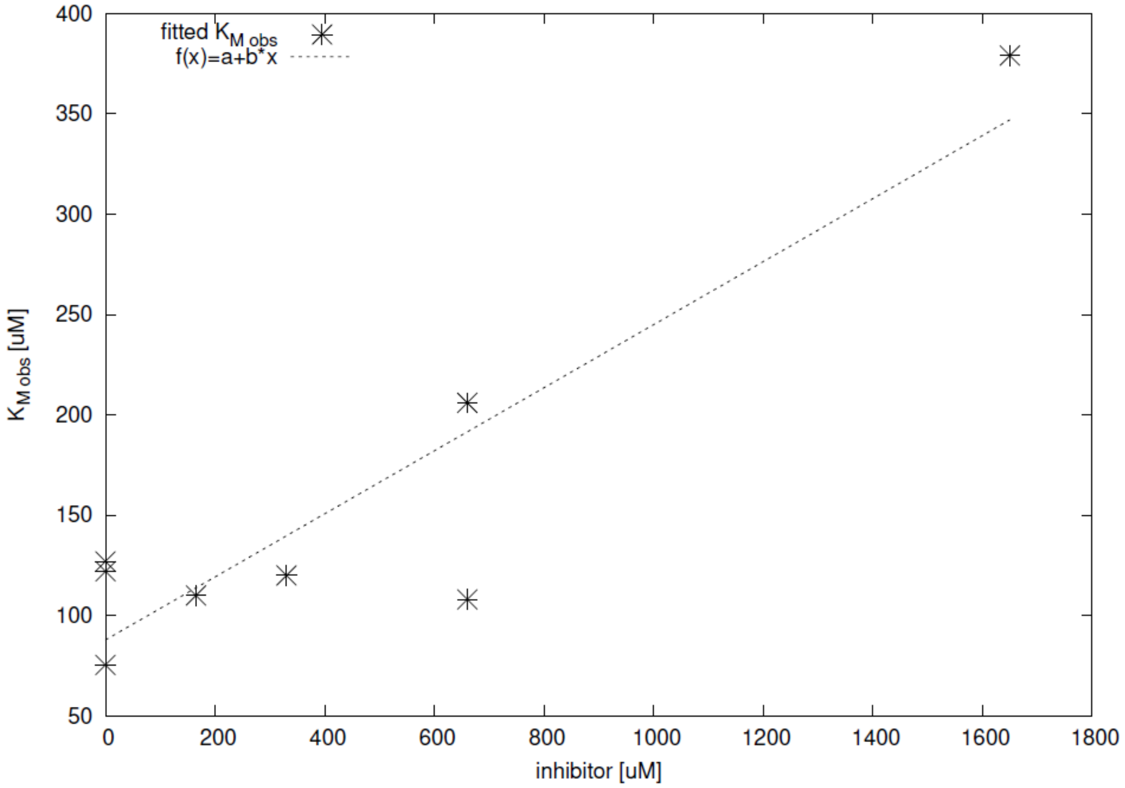

Figure S13. A plot of observed K_M versus various concentration of compound **Va** in hCDA (27nM) catalysed deamination of 2'-deoxycitidine in 100 mM NaCl, 1 mM TCEP, 50 mM phosphate buffer at 25 °C, at pH 4.7.

^{1}H NMR-based kinetic assay of A3-catalysed deamination

Competitive inhibition of A3A mimic and A3A by the synthesised oligonucleotides was performed by a similar procedure reported by us previously [11,12]. We used 400 µM of a standard 7-mer oligonucleotide substrate 5'-dTTTTCAT, 8 or 32 µM of inhibitors, 260 nM of A3A mimic ($A3B_{CTD}$-QM-ΔL3-AL1swap) in a buffer containing 50 mM sodium phosphate (pH 6.0), 100 mM NaCl, 2.5 mM β-mercaptoethanol, 0.1 M EDTA and 50 µM TSP. The H-5 proton doublet signals of the cytosine from the substrate was baselined and integrated. A peak of TSP was used as an internal standard to determine the concentration of the substrate, which appears at 5.88 ppm (J = 7.7 Hz) and converted to 5.71 ppm (J = 8.3 Hz) during the reaction over time. The integrated signal area was converted to substrate concentration and plotted versus time of the reaction. The data were then fitted with linear regression to determine the initial speed of the reaction in the absence of presence of inhibitors.

References

1. Kögler, M.; Busson, R.; De Jonghe, S.; Rozenski, J.; Van Belle, K.; Louat, T.; Munier-Lehmann, H.; Herdewijn, P. *Chem. Biodiv.* **2012**, *9*, 536-556. DOI: 10.1002/cbdv.201100285
2. Gottlieb, H. E.; Kotlyar, V.; Nudelman, A. *J. Org. Chem.* **1997**, *62*, 7512-7515. DOI: 10.1021/jo971176v
3. Mondal, S. Bis(trimethylsiloxy)phosphine. In *e-EROS Encyclopedia of Reagents for Organic Synthesis*, 2013; p https://doi.org/10.1002/047084289X.rn047001561.
4. Xiong, B.; Hu, C.; Gu, J.; Yang, C.; Zhang, P.; Liu, Y.; Tang, K. *ChemistrySelect* **2017**, *2*, 3376-3380. DOI: https://doi.org/10.1002/slct.201700596
5. Kazankova, M. A.; Trostyanskaya, I. G.; Efimova, I. V.; Beletskaya, I. P. *Russ. J. Org. Chem.* **1996**, *32*, 1657-1671.
6. Štimac, A.; Kobe, J. *Carbohydrate Research* **2000**, *329*, 317-324. DOI: https://doi.org/10.1016/S0008-6215(00)00186-5
7. Kolganova, N. A.; Florentiev, V. L.; Chudinov, A. V.; Zasedatelev, A. S.; Timofeev, E. N. *Chem. Biodiv.* **2011**, *8*, 568-576.
8. Harger, M. J. P.; Smith, A. *J. Chem. Soc. Perkin Trans. 1* **1990**, 1447-1451. DOI: 10.1039/p19900001447
9. Shi, K.; Carpenter, M. A.; Kurahashi, K.; Harris, R. S.; Aihara, H. *J. Biol. Chem.* **2015**, *290*, 28120-28130. DOI: 10.1074/jbc.M115.679951
10. Paar, M.; Schrabmair, W.; Mairold, M.; Oettl, K.; Reibnegger, G. *ChemistrySelect* **2019**, *4*, 1903-1908. DOI: 10.1002/slct.201803610
11. Kvach, M. V.; Barzak, F. M.; Harjes, S.; Schares, H. A. M.; Jameson, G. B.; Ayoub, A. M.; Moorthy, R.; Aihara, H.; Harris, R. S.; Filichev, V. V.; et al. *Biochemistry* **2019**, *58*, 391-400. DOI: 10.1021/acs.biochem.8b00858
12. Kvach, M. V.; Barzak, F. M.; Harjes, S.; Schares, H. A. M.; Kurup, H. M.; Jones, K. F.; Sutton, L.; Donahue, J.; D'Aquila, R. T.; Jameson, G. B.; et al. *ChemBioChem* **2020**, *21*, 1028-1035. DOI: 10.1002/cbic.201900505
